# HucMSC-derived exosomes delivered BECN1 induces ferroptosis of hepatic stellate cells via regulating the xCT/GPX4 axis

**DOI:** 10.1038/s41419-022-04764-2

**Published:** 2022-04-08

**Authors:** Youwen Tan, Yan Huang, Rong Mei, Fei Mao, Dakai Yang, Jinwen Liu, Wenrong Xu, Hui Qian, Yongmin Yan

**Affiliations:** 1 The Third Hospital of Zhenjiang Affiliated Jiangsu University, School of Medicine, Jiangsu University, 212003 Zhenjiang, PR China; 2 Key Laboratory of Laboratory Medicine of Jiangsu Province, School of Medicine, Jiangsu University, 212013 Zhenjiang, Jiangsu PR China; 3 The Second Affiliated Hospital of Soochow University, 215004 Suzhou, Jiangsu PR China

**Keywords:** Translational research, Stem-cell research

## Abstract

Activated hepatic stellate cells (HSCs) are significant in liver fibrosis. Our past investigations have shown that human umbilical cord mesenchymal stem cells (hucMSCs) and their secreted exosomes (MSC-ex) could alleviate liver fibrosis via restraining HSCs activation. However, the mechanisms underlying the efficacy were not clear. Ferroptosis is a regulatory cell death caused by excessive lipid peroxidation, and it plays a vital role in the occurrence and development of liver fibrosis. In the present study, we aimed to study the proferroptosis effect and mechanism of MSC-ex in HSCs. MSC-ex were collected and purified from human umbilical cord MSCs. Proferroptosis effect of MSC-ex was examined in HSCs line LX-2 and CCl4 induced liver fibrosis in mice. Gene knockdown or overexpression approaches were used to investigate the biofactors in MSC-ex-mediated ferroptosis regulation. Results: MSC-ex could trigger HSCs ferroptosis by promoting ferroptosis-like cell death, ROS formation, mitochondrial dysfunction, Fe^2^^+^ release, and lipid peroxidation in human HSCs line LX-2. Glutathione peroxidase 4 (GPX4) is a crucial regulator of ferroptosis. We found that intravenous injection of MSC-ex significantly decreased glutathione peroxidase 4 (GPX4) expression in activated HSCs and collagen deposition in experimental mouse fibrotic livers. Mechanistically, MSC-ex derived BECN1 promoted HSCs ferroptosis by suppressing xCT-driven GPX4 expression. In addition, ferritinophagy and necroptosis might also play a role in MSC-ex-promoted LX-2 cell death. Knockdown of BECN1 in MSC diminished proferroptosis and anti-fibrosis effects of MSC-ex in LX-2 and fibrotic livers. MSC-ex may promote xCT/GPX4 mediated HSCs ferroptosis through the delivery of BECN1 and highlights BECN1 as a potential biofactor for alleviating liver fibrosis.

## Introduction

Liver fibrosis is a pathophysiological process caused by chronic viral hepatitis and metabolic disorders. It is characterized by the deposition of many collagen fibers and the proliferation of hepatic stellate cells (HSCs) [1]. Currently, about 500 million patients are suffering from fibrotic liver diseases worldwide. Hepatic fibrosis could cause liver cirrhosis or liver cancers, and there is no effective treatment [2]. Increased activation of HSCs is the main factor leading to the progression of liver fibrosis. Therefore, HSCs are critical targets for ameliorating liver fibrosis [3]. Targeted induction of HSCs inactivation will help establish a more effective anti-fibrosis treatment method.

Mesenchymal stem cells (MSCs) are pluripotent stem cells with self-renewal and multidirectional differentiation capabilities. MSCs have been increasingly applied to clinical treatment, including acute and chronic liver injury [4, 5]. Many clinical trials exploring MSC-based therapy have suggested good advantages in liver disease. However, the potential risks involved in cell therapy are still uncertain [6]. MSC-derived exosomes are membrane vesicles encapsulated with MSC-derived proteins, lipids, mRNAs, and non-coding RNAs [5, 7, 8]. MSC-ex has been proven to play a therapeutic role in eye diseases [9], wound regeneration [10], Parkinson’s disease (PD) [11], and breast cancer [12]. Human bone marrow MSCs (BM-MSC) derived exosomes can inhibit the progression of liver fibrosis by regulating the Wnt/β-catenin signaling pathway [13]. Chorionic plate MSCs (CP-MSC) derived exosomes transferred microRNA-125b inhibits the activation of Hh signaling and promotes liver regeneration [14]. Moreover, umbilical cord MSCs derived exosomes can attenuate inflammation and liver damage in the acute inflammatory liver injury model [15]. In addition, our previous studies have demonstrated that MSC-ex can ameliorate CCl_4_-induced liver fibrosis [16, 17]. These findings suggest that MSC-ex-based therapy can effectively alleviate liver fibrosis, but these curative effect mechanisms are unclear.

Ferroptosis is a new type of programmed cell death that is iron-dependent and different from apoptosis, cell necrosis, and autophagy [18]. Ferroptosis is related to the occurrence and development of many diseases, including Parkinson’s syndrome [19], pancreatic cancer [20], and liver damage [21]. Several studies have demonstrated that ferroptosis induction could ameliorate organizational fibrosis, suggesting a new anti-fibrotic therapy [22]. It has been reported that HSCs ferroptosis could be mediated by chrysophanol [23], magnesium isoglycyrrhizinate [24], or artesunate [25] and thereby alleviated liver fibrosis. However, the role and mechanisms of MSC-ex in regulating HSCs ferroptosis and repairing liver injury is unknown. BECN1 is a crucial regulator of ferroptosis [26], but whether MSC-ex can transport BECN1 to induce HSCs ferroptosis has not been studied.

In the present study, we aimed to investigate the function of MSC-ex in HSCs ferroptosis and the mechanism of action. We observed that MSCs-ex promoted ferroptosis of HSCs. In addition, MSC-ex-derived BECN1 decreased xCT/GPX4 expression and inhibited HSCs activation, providing a novel insight into the proferroptosis effect of MSC-ex in liver fibrosis.

## Materials and methods

### Cell culture

The human umbilical cord was gained from informed, healthy parturients, and MSCs were isolated as previously reported [27]. Isolated MSCs were maintained in α-MEM (Minimum Essential Medium-α) with 10% FBS (fetal bovine serum). Human hepatic stellate cell line LX-2 (#CC4023) was purchased from Saiku Culture Bank (Guangzhou, China) and cultured in H-DMEM (High glucose Dulbecco’s Modified Eagle Medium) with 10% FBS. Human hepatic cell line L-02 cells was purchased from the Chinese Academy of Science and cultured in RPMI 1640 containing 10% FBS at 37°C with 5% CO2. Human fetal lung fibroblasts (HFL-1) were purchased from the Chinese Academy of Sciences and cultured with α-MEM containing 15% FBS at 37 °C with 5% CO2. All the cell lines were authenticated by STR profiling and tested clean for mycoplasma contamination.

### Isolation and characterization of MSC-ex

After reaching 80–90% confluence, the media of MSCs was changed to an FBS-free medium (Bioind) for 48 h and centrifuged at 10,000 × g for 30 min to remove cells and cell debris. MSC-ex was isolated and purified as reported previously [28]. The supernatant collected after that is filtered and sterilized through a 0.22 µm pore filter (Millipore). MSC-ex were then collected and washed with PBS by centrifugation at 10,000 × g for 3 h. Protein concentration of MSC-ex was examined by BCA assay (CWBIO, Beijing, China) and subjected to western blot analysis for CD9, CD63, Calnexin, TSG101, and β-actin. We observed the size and structure of MSC-ex with a transmission electron microscope (TEM) (FEI Tecnai 12; Amsterdam), Atomic Force Microscope (AFM) (Bruker, Germany), and nanoparticle tracking analysis (NTA; NanoSight, UK).

### Western blot

Protein was extracted from MSC-ex, LX-2, or tissues utilizing RIPA (Pierce, Rockford, USA). The concentrations of protein samples were determined by BCA assay (CWBIO, Beijing, China). Proteins were separated in 10% SDS-PAGE denaturing gel and transferred to the nitrocellulose membrane. Next, the membrane was blocked with 5% milk at room temperature. The membrane with blotted protein was incubated with primary antibodies against β-actin (Abclonal, AC026, Wuhan, China), TSG101 (Bioworld, BS91381, CA, USA), Calnexin (Bioworld, BS1438), CD9 (Bioworld, BS3022), CD63 (Abcam, ab271286, UK), Phospho-MLKL (Abclonal, AP0949), LC3B (Abclonal, A19665), GPX4 (Abcam, ab125066), α-SMA (Abcam, ab7817), xCT (Abcam, ab37185), BECN1 (Proteintech, 66665-1-Ig, Chicago, IL, USA). The membrane was further probed with horseradish peroxidase-conjugated secondary antibodies (Invitrogen). Finally, the protein bands were visualized utilizing enhanced chemiluminescence reagents (Millipore).

### qRT-PCR

RNA from LX-2 or liver tissues was collected utilizing Trizol (GIBCO) following the manufacturer’s instructions. cDNA was synthesized from the RNA using SuperScript Reverse Transcriptase Kit (Vazyme, Nanjing, China). Then qRT-PCR assays were performed using SYBR green (CWBIO). Relative gene expression normalized to β-actin was calculated using the 2^−ΔΔCt^ method. All primers were synthesized by Invitrogen and listed in Table 1. There were three replicates per group.

**Table 1:** Primers for Quantitative Real-time PCR.

Genes	Primer Sequence (5′-3′)	Annealing Temperature (°C)	Product size (bp)
Human BECN1	For: GAGGTGAAGAGCATCGGGG	56	302
	Rev: TCTGTGGACATCATCCTGGC		
Human GPX4	For: GTTTTCCGCCAAGGACATCG	55	192
	Rev: TTCCCGAACTGGTTACACGG		
Human xCT	For: TCCTGCTTTGGCTCCATGAACG	58	122
	Rev: AGAGGAGTGTGCTTGCGGACAT		
Human α-SMA	For: CGGACAGCGCCAAGTGAAG	58	97
	Rev: TTGTGTCTAGTTTCTGGGCGG		
Mouse BECN1	For: GCTTACTATTCCTCAGTCCCCT	58	79
	Rev: TTTCTGTAGACATCATCCTAGTCCC		
Mouse GPX4	For: CCGGCTACAACGTCAAGTTT	54	221
	Rev: CGGCAGGTCCTTCTCTATCA		
Mouse xCT	For: ACTGTCACTTTTTGCCCTGGAG	56	329
	Rev: TAAAAAGCCAAGCGCAACCC		
Mouse α-SMA	For: CTATTCCTTCGTGACTACTGCCGAG	58	239
	Rev: TTTCGTGGATGCCCGCTG		
Mouse/Human β-actin	For: CACGAAACTACCTTCAACTCC	56	265
	Rev:CATACTCCTGCTTGCTGATC		

### Immunofluorescence

LX-2 was planted on coverslips in a 24-well plate and treated with PBS or MSC-ex (400 or 800 μg/ml). After MSC-ex treatment, LX-2 was fixed in 4% (w/v) paraformaldehyde (Sigma-Aldrich) for 15 min. LX-2 was then permeabilized with 0.1% Triton X-100 for 10 min. 5% BSA was utilized to block the cells at room temperature for 1 h. After that, LX-2 was hybridized with primary antibodies against α-SMA, GPX4, and xCT (Abcam) at 4 °C overnight and visualized with fluorescence-labeled secondary antibodies (Abclonal) for 45 min at 37 °C. Hoechst 3342 was used to stain nuclei at room temperature. Images were captured using a confocal microscope (Nikon, Tokyo).

### Ethics statement

All experiments involving animals were conducted according to the ethical policies and procedures approved by the ethics committee of the Jiangsu University ethics committee (Approval no. UJS-IACUC-AP-2020033127). The use of human umbilical cord tissues was approved by the Jiangsu University ethics committee (2012258).

### Mouse model of liver fibrosis and MSC-ex injection

Mice (BALB/c, female, 4–5 weeks) were from Cavens animal laboratory Co., Ltd from Changzhou, China. Following the protocols approved by the Jiangsu University ethics committee (UJS-IACUC-AP-2020033127), a mouse model of liver fibrosis was established randomly with an intraperitoneal injection of 10% CCl4 (dissolved in mineral oil, 0.1 ml/100 g body weight) for eight weeks, twice a week. To analyze the effect of MSC-ex on GPX4, BECN1, and xCT expression and liver fibrosis, mice were injected with MSC-ex twice a week at a dose of 20 mg/kg body weight (*n* = 6) or PBS (*n* = 6). Liver samples were collected for further analysis at two weeks post-MSC-ex injection.

### Sirius red staining

According to the manufacturer’s instruction, the manufacturer’s instruction measured collagen deposition in liver sections in Sirius Red (Chondrex, USA). To analyze hepatic collagen distribution, fibrotic septa randomly selected from the right and left liver lobes of 6 individual mice/groups were assessed. Images were acquired using a digital slide scanner (3DHISTECH, Hungary). Collagen extent was expressed as a percentage of stained area in each liver section.

### Immunohistochemistry

The liver sections were deparaffinized and rehydrated for immunohistochemical staining for BECN1 and GPX4; the endogenous peroxidase was inactivated by 3% H2O2 for 30 min. Next, the liver slides were immersed into preheated antigen retrieval solution (0.01 M, pH 6.0, citrate buffer) for 30 min. After blocking with 5% BSA, the liver slides were probed with primary antibody against BECN1 (Proteintech) and GPX4 (Abcam) overnight at 4 °C and incubated with biotin-conjugated anti-rabbit IgG and streptavidin-biotin. Finally, liver tissue sections were visualized with DAB Horseradish Peroxidase Color Development Kit (Boster, Wuhan, China) and counterstained with hematoxylin. Images were acquired using a pathological section scanner (3DHISTECH, Hungary).

### FDA assay

MSC-ex, MSC-ex/NEC-1 or NEC-1s (necrostatin-1, MedChemExpress), MSC-ex/NAC (N-acetyl-L-cysteine, Sigma) or Erastin (MedChemExpress) treated LX-2 or L-02 were cultured in medium supplemented with 10 µM FDA at 37 °C for 20 min. Finally, the cell viability of MSC-ex treated LX-2, or L-02, was detected by Cytation 5 Imaging Reader (Biotek, Vermont, USA). There were three replicates per group.

### ROS assay

MSC-ex, MSC-ex/NEC-1 or NEC-1s, MSC-ex/NAC (N-acetyl-L-cysteine, Sigma) or Erastin (MedChemExpress) treated LX-2 or L-02 were cultured in medium supplemented with 10 mM fluorescent probe DCFH-DA (Beyotime, Shanghai, China) for 20 min at 37 °C. After fixing with 4% paraformaldehyde, LX-2 or L-02 were counterstained with Hoechst 3342. Images were captured using a confocal microscope (Nikon, Tokyo, Japan). Mean fluorescent intensity was calculated using Image-Pro Plus 6.0 software. There were three replicates per group.

### Mitochondrial membrane potential assay

JC-1 staining was performed to analyze the mitochondrial membrane potential in LX-2 or L-02 (Beyotime, Shanghai, China). JC-1 probe was diluted in fresh medium at 1:1000. Cells were incubated with a diluted JC-1 probe in the dark at 37 °C for 20 min. After fixing with 4% paraformaldehyde, LX-2 or L-02 were counterstained with Hoechst 3342. Images were captured using a confocal microscope (Nikon, Tokyo, Japan). Mean fluorescent intensity was calculated using Image-Pro Plus 6.0 software. There were three replicates per group.

### Iron concentration assay

MSC-ex (400 or 800 μg/ml) or *BECN1* cDNA treated LX-2 or L-02 were lysed utilizing RIPA (Pierce, Rockford, USA), and iron concentrations were determined using an iron assay kit following the manufacturer’s instructions (Abcam). There were three replicates per group.

### GSH and MDA quantification analysis

Levels of GSH and MDA in MSC-ex (400 or 800 μg/ml) treated LX-2 or L-02 were detected with GSH assay kit (Beyotime, Shanghai, China) and MDA assay kit (Beyotime, Shanghai, China) following the manufacturer’s instructions, respectively. There were three replicates per group.

### Transmission electron microscopy (TEM) analysis of mitochondrial ultrastructure

PBS or MSC-ex (800 μg/ml) treated LX-2 was plated in 60 mm culture dishes. For intracellular mitochondrial ultrastructure observation, LX-2 was collected and fixed with 2.5% glutaraldehyde in 3 mM CaCl2 and 0.1 M cacodylate. After cutting and staining with uranyl acetate and lead citrate, images of ultrathin sections were acquired with a transmission electron microscope (FEI Tecnai 12, Philips, Netherlands).

### *BECN1* cDNA and siRNA transfection

Full-length human *BECN1* cDNA (NM_003766) was constructed into pBECN1 expression vectors (pCDH-CMV-pBECN1-EF1a-GFP-T2A-Puro). Small interfering RNA (siRNA) targeting BECN1 and control scrambled siRNA were designed (Genepharma, Suzhou, China). LX-2 was seeded to 80% confluence in 6-well plates in triplicate. Lipofectamine 2000 (Life Technologies) reagent was used to transfigure BECN1 expression vector or synthetic small interfering RNA (siRNA) oligos into LX-2. Cell protein or RNA samples were harvested at 48 h after transfection and processed for western blot or Quantitative RT-PCR analysis.

### Lentiviral knockdown of BECN1 in MSCs

Recombinant lentiviruses were produced by transient transfection in HEK293T cells. BECN1 shRNA sequences were synthesized and cloned into pPLK/GFP-Puro lentiviral plasmid to generate pPLK/GFP-Puro-BECN1 shRNA/control shRNA. Sequences of BECN1 shRNA are designed using the following: GTGGACACGAGTTT CAAGA. Then HEK293T cells were co-transfected with three lentiviral plasmids of psPAX2 (packaging vector), pMD2G (envelope vector), and pPLK/GFP-Puro-BECN1 shRNA (transfer vector). GFP expression of HEK293T cells was evaluated, and supernatants were collected at 72 h post-co-transfection. MSCs were transfected with lenti-BECN1-shRNA/lenti-control-shRNA lentivirus. BECN1 expression in MSCs was examined with qRT-PCR and western blot. Exosomes of lenti-BECN1 shRNA transfected MSCs (Ex^shBECN1^) or lenti-control shRNA transfected MSCs (Ex^sh-ctr^) were collected and purified. To investigate the regulation of Ex^sh-ctr^ and Ex^shBECN1^ on GPX4, mice were injected with PBS (*n* = 6), Ex^sh-ctr^ (*n* = 6), and Ex^shBECN1^ (*n* = 6) at a dose of 20 mg/kg body weight twice a week. At two weeks post Ex^shBECN1^ injection, liver samples were collected for further analysis.

### Statistical analysis

Data are presented as means ± SD (standard deviation). The student’s t-test used GraphPad Prism version 5.0 software to analyze statistical significance between two groups. One-way analysis of variance (*ANOVA*) followed with Dunnett was used for studies involving more than two groups. A two-sided *P* < 0.05 was considered statistically significant.

## Results

### MSC-ex induces HSCs ferroptosis in vitro

**Fig. 1:**
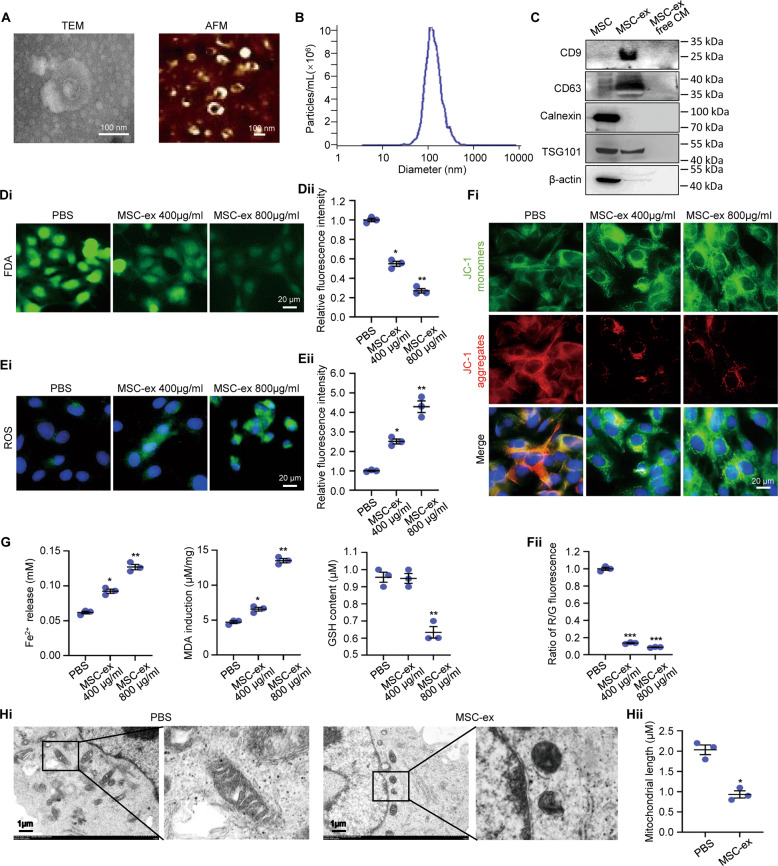
MSC-ex induced LX-2 ferroptosis in vitro. **A** Representative images of the nano-size vesicles photographed by transmission electron microscope and atomic force microscope. Scale bar, 100 nm. **B** Nanoparticle tracking analyses of the MSC-ex in PBS. **C** Exosome markers (CD9, CD63, TSG101) and non-exosome markers of Calnexin detection by western blot analysis. **D** FDA staining of cell death in MSC-ex (400, 800 µg/ml) treated LX-2 (*n* = 3; * *P* < 0.05 compared with PBS group, **P < 0.01 compared with PBS group). Scale bar, 500 μm. **E** ROS production in MSC-ex (400, 800 µg/ml) treated LX-2 by DCF probe staining (*n* = 3; * *P* < 0.05 compared with PBS group, ** *P* < 0.01 compared with PBS group). Scale bar, 20 μm. **F** Mitochondria membrane potential in MSC-ex (400, 800 µg/ml) treated LX-2 by JC-1 staining (*n* = 3; *** *P* < 0.001 compared with PBS group). Scale bar, 20 μm. G The Fe2+ release, MDA induction and GSH level in MSC-ex (400, 800 µg/ml) treated LX-2 (*n* = 3; * *P* < 0.05 compared with PBS group, ** *P* < 0.01 compared with PBS group). **H** Mitochondrial structure of MSC-ex (800 µg/ml) treated LX-2 were observed by transmission electron microscopy (*n* = 3; * *P* < 0.05 compared with PBS group). MSC-ex Mesenchymal stem cell derived exosome. PBS Phosphate buffered saline. ROS Reactive oxygen species. FDA Fluorescein Diacetate. MDA Malondialdehyde. GSH Glutathione, r-glutamyl cysteingl glycine. DCF, 2’,7’Dichlorofluorescein.

Ferroptosis is closely related to the occurrence and development of chronic liver disease, liver fibrosis, and liver cancer [18]. HSCs ferroptosis has been targeted for inhibiting liver fibrosis [24]. Therefore, we hypothesized that MSC-ex ameliorates fibrosis through modulating ferroptosis of HSCs. To test this hypothesis, isolated MSC-ex were characterized and incubated with HSCs line LX-2. The purified MSC-ex displayed a round spherical shape of around 100 nm, and the mean particle concentration is about 1 × 10^7^ particles/ml (Fig.1A, B). Western blot was used to investigate exosome markers CD9, CD63, TSG101, and ER membrane marker Calnexin (Fig. 1C, Supplemental Fig. 9A).

Then, 400 μg/ml or 800 μg/ml of MSC-ex were added to cultured LX-2 for 48 h. FDA staining showed that MSC-ex could induce significant cell death in LX-2, as demonstrated by decreased green fluorescence intensity in the MSC-ex group (Fig. 1D). Next, we investigated whether MSC-ex caused LX-2 ferroptosis by measuring ferroptosis-related markers. Intracellular ROS levels, mitochondrial membrane potential, Fe^2+^ level, lipid peroxides malondialdehyde (MDA), glutathione (GSH), and mitochondria morphology were determined to observe the level of cell ferroptosis. As expected, MSC-ex induced ferroptosis was revealed by increased intracellular ROS production (Fig. 1E), reduced mitochondrial membrane potential (Fig. 1F), excessive Fe^2+^, lipid peroxides, and decreased GSH (Fig. 1G). Furthermore, MSC-ex enhanced cell death, ROS production, and decreased mitochondrial membrane potential ferroptosis was reverted by ferroptosis inhibitors NAC addition (Supplemental Fig. 1A–C). In addition, transmission electron microscopy images of mitochondrial structure demonstrated induced LX-2 ferroptosis in the MSC-ex group. MSC-ex treated LX-2 showed crumpled mitochondria with increased membrane density than the PBS group (Fig. 1H).

**Fig. 2:**
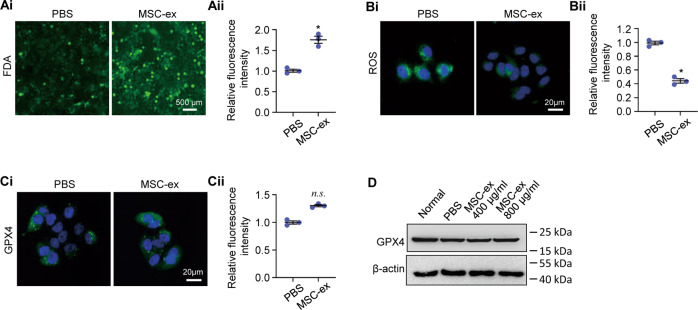
MSC-ex didn’t affect hepatocyte ferroptosis in vitro. **A** FDA staining of cell death in MSC-ex treated L-02 cells (*n* = 3; * *P* < 0.05 compared with PBS group). Scale bar, 500 μm. **B** ROS production in MSC-ex treated L-02 cells by DCF probe staining (*n* = 3; *P < 0.05 compared with PBS group). Scale bar, 20 μm. **C** Representative images of immunofluorescence staining for GPX4 in MSC-ex (800 µg/ml) treated human hepatocyte cell line L-02 (*n* = 3; n.s. Not significant, compared with PBS group). Scale bar, 20 μm. **D** Western blot analysis of GPX4 expression in MSC-ex (400, 800 µg/ml) treated L-02. MSC-ex Mesenchymal stem cell-derived exosome. PBS Phosphate-buffered saline. ROS Reactive oxygen species. DCF, 2’,7’ Dichlorofluorescein. GPX4 Glutathione peroxidase 4.

To elucidate the specificity of MSC-ex on LX-2 cell death, the effects of MSC-ex on human hepatic cell line L-02 and human fetal lung fibroblast (HFL-1) ferroptosis were also investigated with FDA assay and ROS assay. The FDA and ROS assay results showed that L-02 cell death and ROS production were decreased by MSC-ex (Fig. 2A, B). MSC-ex exerted an inhibitory effect of L-02 ferroptosis. Expression of ferroptosis marker glutathione peroxidase 4 (GPX4) was also not changed in MSC-ex treated L-02 (Fig. 2C, D, Supplemental Fig. 9B). MSC-ex treatment did not induce cell death and ROS production in HFL-1 (Supplemental Fig. 2). In conclusion, these data showed that MSC-ex caused HSCs ferroptosis in vitro.

### MSC-ex-derived BECN1 downregulates System xc-/GPX4 expression in LX-2

**Fig. 3:**
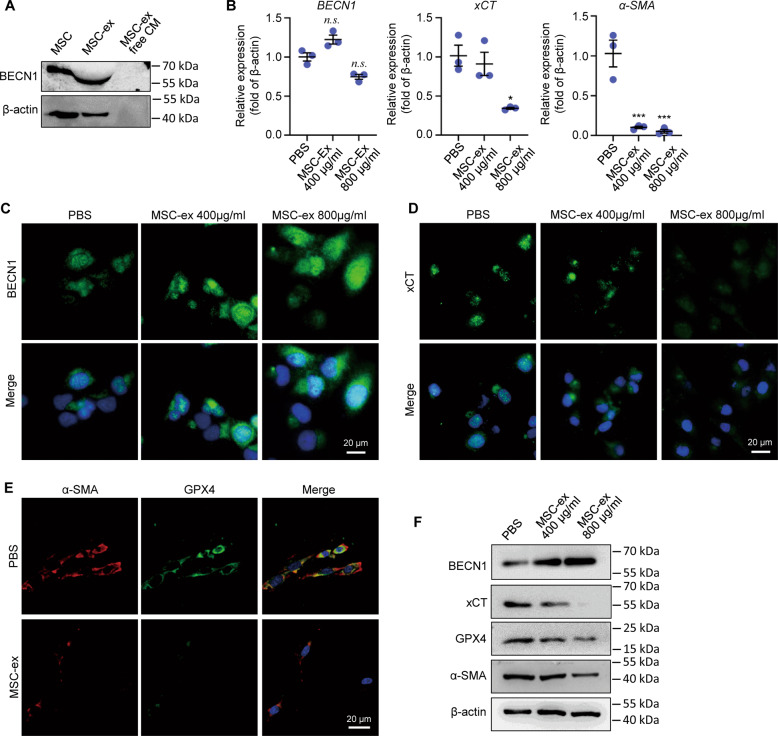
MSC-ex delivered BECN1 upregulated System xc-/GPX4 axis in LX-2. **A** Western blot quantification of BECN1 expression in MSC, MSC-ex and MSC-ex free conditioned medium (CM). **B** mRNA expression of BECN1, xCT and α-SMA in MSC-ex (400, 800 µg/ml) treated LX-2 at 48 h measured by qRT-PCR (*n* = 3; *P < 0.05 compared with PBS group, ***P < 0.001 compared with PBS group; n.s. Not significant, compared with PBS group). **C**–**E** Representative images of BECN1, xCT, GPX4 and α-SMA protein expression in MSC-ex (400, 800 µg/ml) treated LX-2 by immunofluorescence. Scale bars, 20 μm. **F** Western blot analysis of BECN1, xCT, GPX4 and α-SMA expression in MSC-ex (400, 800 µg/ml) treated LX-2. MSC-ex Mesenchymal stem cell-derived exosome. PBS Phosphate buffered saline. GPX4 Glutathione peroxidase 4. α-SMA Alpha-smooth muscle actin. System xc Cystine/glutamate antiporter system. xCT Cystine/glutamate exchange transporter.

xCT and SLC7A11 are components of the xc system amino acid antiporter, which import cysteine and export glutamate into cells [18]. BECN1 induces ferroptosis through binding to xCT and blocking xc- activity [29]. Thus, we hypothesized that BECN1 could initiate the proferroptosis effect of MSCs-Ex. We found that BECN1 was enriched both in MSCs and MSC-ex, whereas not observed in MSC-ex-free CM (Fig. 3A, Supplemental Fig. 9C). Downregulated xCT/GPX4 signaling contributes to ferroptosis, which is necessary to activate HSCs. Then, expression of BECN1, xCT, GPX4, and HSCs activation marker a-SMA were detected in MSC-ex treated LX-2. BECN1 mRNA level was not changed (Fig. 3B), whereas BECN1 protein was significantly increased (Fig. 3C, F, Supplemental Fig. 3, Supplemental Fig. 9D). Furthermore, expression of xCT and α-SMA mRNA in MSC-ex treated LX-2 decreased compared with PBS control (Fig. 3B). Similarly, immunofluorescence and western blot analysis confirmed that MSC-ex could downregulate xCT, GPX4, and a-SMA protein expression (Fig. 3D–F, Supplemental Figs. 3, 9D). These results show that MSC-ex may induce HSCs ferroptosis and inactivation through exosomes mediated BECN1 protein transfer but not BECN1 mRNA regulation.

### MSC-ex induces BECN1 mediated ferroptosis in mouse fibrotic liver

**Fig. 4:**
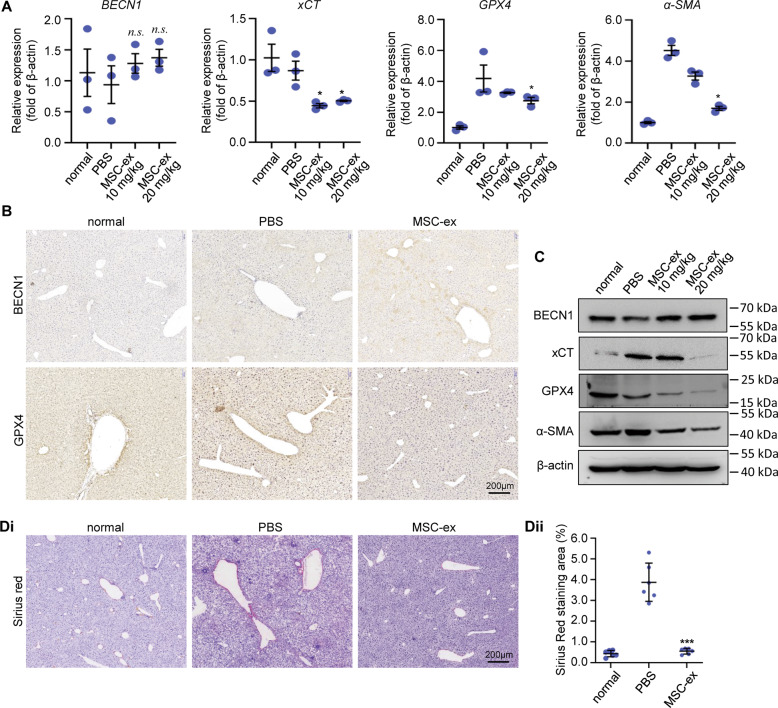
MSC-ex upregulated BECN1/System xc-/GPX4 axis and inhibited collagen deposition in mouse fibrotic liver. **A** mRNA expression of BECN1, xCT, GPX4 and α-SMA in MSC-ex (10, 20 mg/kg) treated mice livers was measured by qRT-PCR (*n* = 3; *P < 0.05 compared with PBS group; n.s. Not significant, compared with PBS group). **B** Representative images of immunohistochemical staining for BECN1 and GPX4 staining in MSC-ex (20 mg/kg) treated mice livers. **C** Western blot analysis of BECN1, xCT, GPX4 and α-SMA expression in MSC-ex (10, 20 mg/kg) treated mice livers. **D** Sirius Red staining of collagen deposition in MSC-ex (20 mg/kg) treated mice livers (*n* = 6; ***P < 0.001 compared with PBS group). MSC-ex Mesenchymal stem cell-derived exosome. GPX4 Glutathione peroxidase 4. α-SMA Alpha smooth muscle actin. System xc- Cystine/glutamate antiporter system. xCT Cystine/glutamate exchange transporter.

Our previous report showed that MSC-ex could significantly decrease liver fibrosis by downregulating HSCs activation [16]. To investigate the mechanisms of the MSC-ex mediated proferroptosis effect, we speculated that MSC-ex delivered BECN1 induced ferroptosis of activated HSCs. Then the expression of ferroptosis- and HSCs activation-associated biomarkers such as BECN1, xCT, GPX4, and a-SMA were examined in liver tissues from MSC-ex-treated mice. Consistent with the expression of BECN1 mRNA in MSC-ex treated LX-2 (Fig. 3B), qRT-PCR results showed that MSC-ex also did not affect BECN1 mRNA expression in fibrotic livers (Fig. 4A). xCT, GPX4, and a-SMA mRNA levels were downregulated in MSC-ex (10 or 20 mg/kg) treated mouse fibrotic livers (Fig. 4A). Immunohistochemistry staining assay and western blot analysis revealed that BECN1 level was increased in MSC-ex (10 or 20 mg/kg) treated mouse fibrotic liver compared to which in CCl_4_ group (Fig. 4B, C, Supplemental Figs. 4, 9E). xCT, GPX4, and a-SMA were cumulated in fibrotic livers and were decreased by MSC-ex (Fig. 4B, C, Supplemental Fig. 4). Furthermore, Sirius red staining revealed that MSC-ex reduced collagen deposition in fibrotic livers (Fig. 4D). Thus, MSC-ex containing BECN1 may induce BECN1/System xc-/GPX4 mediated ferroptosis and reduce HSCs activation in mouse fibrotic livers.

### BECN1 induces LX-2 ferroptosis through downregulating System xc-/GPX4

**Fig. 5:**
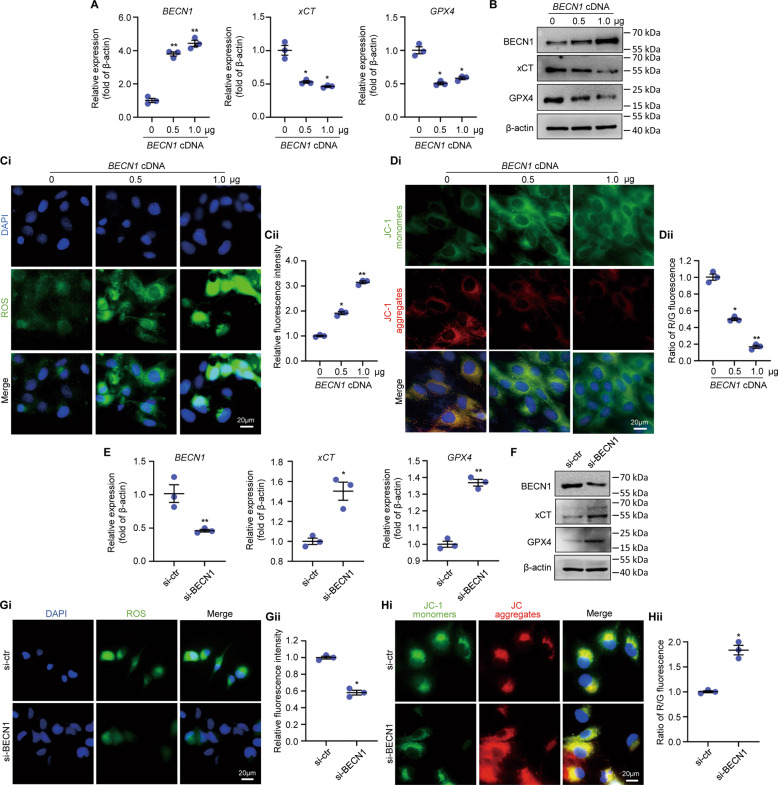
BECN1 regulated LX-2 ferroptosis in vitro. **A** mRNA expression of BECN1, xCT and GPX4 in LX-2 transfected with 0.5 or 1.0 µg *BECN1* cDNA (*n* = 3; *P < 0.05 compared with *BECN1* cDNA 0 μg group, **P < 0.01 compared with *BECN1* cDNA 0 μg group). **B** Western blot analysis of BECN1, xCT and GPX4 expression in LX-2 transfected with 0.5 or 1.0 µg *BECN1* cDNA. **C** ROS production in LX-2 by DCF probe staining (*n* = 3; *P < 0.05 compared with *BECN1* cDNA 0 μg group, **P < 0.01 compared with *BECN1* cDNA 0 μg group). Scale bar, 20 μm. **D** Mitochondria membrane potential in LX-2 by JC-1 staining (*n* = 3; *P < 0.05 compared with *BECN1* cDNA 0 μg group, **P < 0.01 compared with *BECN1* cDNA 0 μg group). Scale bar, 20 μm. **E** mRNA expression of BECN1, xCT and GPX4 in LX-2 transfected with control siRNA (si-ctr) or BECN1 siRNA (siBECN1) (n = 3; *P < 0.05 compared with si-ctr group, **P < 0.01 compared with si-ctr group). **F** Western blot analysis of BECN1, xCT and GPX4 expression in LX-2 transfected with control siRNA (si-ctr) or BECN1 siRNA (siBECN1). **G** ROS production in LX-2 by DCF probe staining (*n* = 3; *P < 0.05 compared with si-ctr group). Scale bar, 20 μm. **H** Mitochondria membrane potential in LX-2 by JC-1 staining (*n* = 3; *P < 0.05 compared with si-ctr group). Scale bar, 20 μm. MSC-ex Mesenchymal stem cell derived exosome. GPX4 Glutathione peroxidase 4. xCT Cystine/glutamate exchange transporter. ROS Reactive oxygen species. DCF, 2’,7’ Dichlorofluorescein.

To further evaluate the effects of BECN1 in the induction of HSCs ferroptosis, we altered BECN1 expression in LX-2 using recombinant BECN1 cDNA or BECN1 siRNA transfection. We found that BECN1 cDNA transfection significantly increased BECN1 mRNA and protein expression and decreased xCT and GPX4 mRNA and protein expression dose-dependently in LX-2 (Fig. 5A, B, Supplemental Figs. 5A, 9F). Promoted ROS production and declined mitochondrial membrane potential were stimulated in BECN1 cDNA (0.5 or 1.0 µg/ml) transfected LX-2 (Fig. 5C, D). These data imply that BECN1 overexpression can downregulate System xc-/GPX4 expression and induce LX-2 ferroptosis. To consolidate the proferroptosis effect of BECN1, we knocked down BECN1 in LX-2 with BECN1 siRNA. As expected, BECN1 knockdown increased xCT/GPX4 expression in LX-2 (Fig. 5E, F, Supplemental Fig. 5B, Supplemental Fig. 9G). ROS production was decreased, and mitochondrial membrane potential was increased by BECN1 knockdown (Fig. 5G, H). Thus, BECN1 can induce LX-2 ferroptosis through downregulating System xc-/GPX4.

Ferritinophagy, a selective form of autophagy, contributes to the initiation of ferroptosis through degradation of ferritin, which triggers iron overload (IO), lipid peroxidation, membrane damage, and cell death [30]. To further explore the mechanism of MSC-ex regulating ferroptosis, we analyzed the effect of MSC-ex on LX-2 autophagy. MSC-ex promoted autophagy markers LC3B expression in LX-2 cells (Supplemental Figs. 6A, B, 9K). In addition to regulating XCT expression, BECN1 is also an autophagy initiation factor and has a central role in autophagy. We found that Beclin-1 overexpression improved autophagy markers LC3BII expression (Supplemental Figs. 6C, 9L) and intracellular Fe^2+^ level in LX-2 cells (Supplemental Fig. 6D). These results indicated that MSC-ex might also promote ferroptosis through exosomal BECN1 induced ferritinophagy.

### BECN1 knockdown attenuated the effect of MSC-ex on LX-2 ferroptosis

**Fig. 6:**
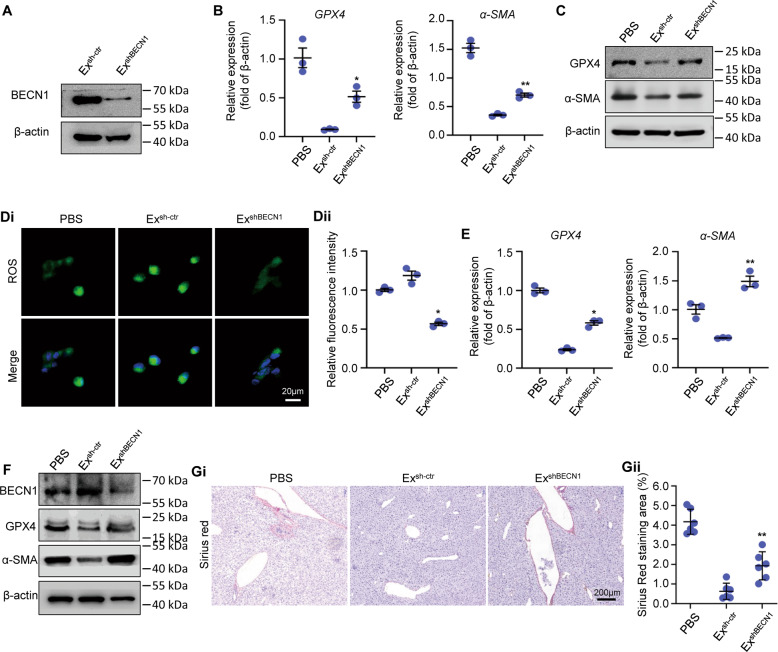
BECN1 knockdown attenuated the effect of MSC-ex on LX-2 ferroptosis. **A** Western blot analysis of BECN1 in exosomes from MSCs transfected with control shRNA (Ex^sh-ctr^) or BECN1-shRNA (Ex^shBECN1^). **B** mRNA expression of GPX4 and α-SMA in Ex^sh-ctr^ or Ex^shBECN1^ treated LX-2 (*n* = 3; *P < 0.05 compared with Ex^sh-ctr^ group, **P < 0.01 compared with Ex^sh-ctr^ group). **C** Western blot analysis of GPX4 and α-SMA expression in Ex^sh-ctr^ or Ex^shBECN1^ treated LX-2. **D** ROS production in Ex^sh-ctr^ or Ex^shBECN1^ treated LX-2 by DCF probe staining (*n* = 3; *P < 0.05 compared with Ex^sh-ctr^ group). Scale bar, 20 μm. **E** mRNA expression of GPX4 and α-SMA in livers from Ex^sh-ctr^ (20 mg/kg) or Ex^shBECN1^ (20 mg/kg) treated mice (*n* = 3; *P < 0.05 compared with Ex^sh-ctr^ group, **P < 0.01 compared with Ex^sh-ctr^ group). **F** Western blot analysis of BECN1, GPX4, and α-SMA expression in livers from Ex^sh-ctr^ (20 mg/kg) or Ex^shBECN1^ (20 mg/kg) treated mice. **G** Sirius Red staining of collagen deposition in livers from Ex^sh-ctr^ (20 mg/kg) or Ex^shBECN1^ (20 mg/kg) treated mice (*n* = 6; **P < 0.01 compared with Ex^sh-ctr^ group). ROS Reactive oxygen species. DCF, 2’,7’ Dichlorofluorescein. α-SMA Alpha smooth muscle actin.

To further elucidate the role of MSC-ex derived BECN1 in ferroptosis induction and liver fibrosis amelioration, we compared the role of MSC-ex with shRNA control (Ex^sh-ctr^) or BECN1 knockdown (Ex^shBECN1^) in the regulation of GPX4 and a-SMA expression, ROS production, and hepatic collagen deposition in vitro and in vivo. Compared with Ex^sh-ctr^, the level of BECN1 was decreased in Ex^shBECN1^ (Fig. 6A, Supplemental Fig. 9H). GPX4 and a-SMA downregulation by MSC-ex treatment in LX-2 was reversed by BECN1 knockdown (Fig. 6B, C, Supplemental Fig. 9I). Increased ROS production in MSC-ex treated LX-2 was reversed by BECN1 knockdown (Fig. 6D). Furthermore, MSC-ex mediated GPX4 and a-SMA down-regulation was also changed by BECN1 knockdown in fibrotic livers (Fig. 6E, F, Supplemental Fig. 9J). BECN1 knockdown also attenuated inhibition of collagen deposition of MSC-ex on CCl_4_-induced liver fibrosis (Fig. 6G). Thus, BECN1 knockdown delayed MSC-ex induced HSCs ferroptosis, so BECN1 plays a crucial role in MSC-ex mediated proferroptosis effect.

Finally, we explored whether MSC-ex regulates LX-2 cell death through holding necroptosis. MLKL is considered a central executor of necroptosis, and phosphorylation of MLKL (p-MLKL) was a critical event of necroptosis [31]. MSC-ex treatment significantly increased p-MLKL expression in LX-2 cells, which was reverted by Necroptosis inhibitor NEC-1 or NEC-1s (Supplemental Figs. 7A, B; 8A, 9M, N). Furthermore, MSC-ex mediated LX-2 cell death, and ROS production was attenuated by necroptosis inhibitor NEC-1 or NEC-1s (Supplemental Figs. 7C, D, 8C, D). These results indicate that MSC-ex may induce necroptosis in LX-2 cells.

## Discussion

This study found MSC-ex-derived BECN1 induced HSCs ferroptosis and ameliorated liver fibrosis. In vitro, MSC-ex increased BECN1 expression in LX-2 through exosomal BECN1 transfer and subsequently inhibited the expression of xCT and GPX4 in HSCs. Then the word of α-SMA in LX-2 was decreased following ferroptosis. Our results showed that overexpression of BECN1 in LX-2 enhanced ferroptosis, whereas knockdown of BECN1 in LX-2 abolished ferroptosis, further suggesting that BECN1 was crucial in HSCs ferroptosis induction. In vivo, the ability of MSC-ex to induce ferroptosis and ameliorate liver fibrosis was weakened following BECN1 knockdown. Briefly, MSC-ex could enhance ferroptosis in HSCs and subsequently reduce CCl4-induced liver injury through exosomes/ BECN1/xCT/GPX4 pathway.

Liver fibrosis is a progressive disease. MSC-ex exerted therapeutic effects in mouse liver fibrosis. However, the mechanism of MSC-ex reducing liver fibrosis remains obscure. It has been confirmed that ferroptosis is essential for defending against various liver diseases, such as liver fibrosis [18], hepatocellular carcinoma [32], ischemia-reperfusion liver injury [33], and acetaminophen-induced liver injury [34]. HSCs are essential for the pathogenesis of liver fibrosis. Thus, targeting HSCs ferroptosis is considered a therapeutic approach to alleviate liver fibrosis. In MSC-ex treated LX-2, ferroptosis markers such as cell death, intracellular ROS production, and GPX4 expression were increased. However, the proferroptosis effect of ROS production improvement and GPX4 expression downregulation of MSC-ex was not found on human hepatocyte cell line L-02, which was in line with previous reports [17]. We discovered that MSC-ex significantly downregulated xCT expression in LX-2 cells but not L-02 cells. xCT is a membrane antiporter that exports intracellular glutamate in exchange for extracellular cysteine, thereby protecting cells from oxidative stress and ferroptosis [35]. Recent research found that xCT mRNA levels were log-fold lower in primary mouse hepatocytes than in primary mouse HSCs. Inhibiting xCT in LX-2 cells by Erastin induced massive ferroptosis in HSCs did not affect hepatocytes [36]. Thus, we reasoned that MSC-ex-mediated xCT inhibition might preferentially promote LX-2 ferroptosis but not L-02 hepatocytes. L-02 cells were resistant to the treatment with MSC-ex, whereas the LX-2 cells were sensible.

Exosomes contain complex RNAs and proteins and have aroused widespread interest in the regeneration and repair of various tissues [5, 8, 37]. Evidence suggests that MSC has therapeutic potential in liver fibrosis. However, whether MSC-ex can induce ferroptosis remains unclear. We aimed to investigate the critical ferroptosis components present in MSC-ex. BECN1 is an important ferroptosis that can regulate various cellular processes [38]. Our findings confirmed that BECN1 was enriched in MSC-ex. Overexpression and knockdown of BECN1 in LX-2 suggested that BECN1 played crucial roles in ferroptosis and inactivation of HSCs. In vivo, we knocked down BECN1 in MSC-ex and confirmed the part of MSC-ex-derived BECN1 in ferroptosis and anti-fibrotic effects. These results indicated that MSC-ex exosomal BECN1 might be crucial for MSC-ex-mediated HSCs ferroptosis and liver fibrosis treatment. We compared BECN1 expression in MSC-ex treated LX-2 cells, and recombinant BECN1 cDNA transfected LX-2. As shown in Figs. 3F and 5B, BECN1 presentation in the MSC-ex 800 μg/ml group increased by 2.86 times, while the 1 μg BECN1 cDNA group increased by 3.01 times. Furthermore, xCT mRNA and protein expression in both MSC-ex 800 μg/ml group (Fig. 3A, F) and BECN1 cDNA 1 μg group (Fig. 5A, B) was significantly suppressed. MSC-exosomal BECN1 may suppress xCT activity through transcriptional inhibition. Thus MSC-ex treatment can correspond to the amounts of BECN1 levels after overexpression.

We further explored the underlying mechanisms by which MSC-ex derived BECN1 can induce ferroptosis and present hepatoprotective effects. BECN1, xCT, and GPX4 inhibition are all reported to trigger ferroptosis [39]. BECN1 can inhibit system xc-activity and induce lipid peroxidation through binding to xCT [38]. We then examined BECN1, xCT, and GPX4 expression in MSC-ex treated LX-2 and mouse fibrotic livers. BECN1 was significantly increased, whereas xCT and GPX4 expression were decreased in vitro and in vivo. Alteration of BECN1 affects xCT and GPX4 expression and ferroptosis. Our results suggested that MSC-exosomal BECN1 may promote ferroptosis by reducing the xCT expression. BECN1 is an autophagy initiation factor and has a central role in autophagy [26]. We found that BECN1 overexpression and MSC-ex upregulated autophagy marker LC3B expression promoted Fe2+ release in LX-2 cells. These results indicated that MSC-exosomal BECN1 might promote ferroptosis by inducing ferritinophagy. Furthermore, MSC-ex mediated LX-2 cell death, and ROS production was also attenuated by necroptosis inhibitor NEC-1. However, the exact role and mechanisms of MSC-ex in ferritinophagy and necroptosis regulation in HSCs need to be further elucidated.

Finally, NAC has significant protective activity against liver fibrosis through attenuation of oxidative stress. Our previous study also showed that MSC-ex could act as an antioxidant to reduce oxidative stress and rescue liver failure in CCl4 induced liver injury mouse model [8, 17]. Our results showed that MSC-ex was a ferroptosis inducer to inactivate HSCs in vitro and vivo. Thus, we reasoned that combining the antioxidant effect of NAC and the proferroptotic effect of MSC-ex may produce a better efficacy in liver disease associated with fibrosis. However, NAC also interferes with the proferroptotic development of MSC-ex. The specific dose and effectiveness need to be further explored.

## Conclusion

**Fig. 7:**
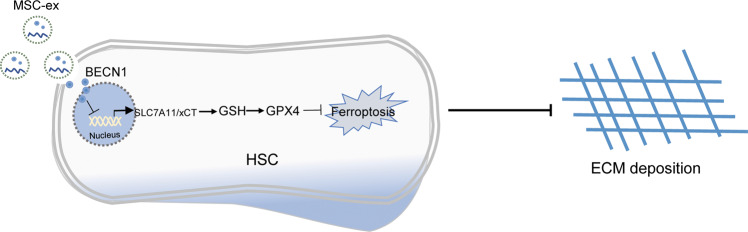
Schematic representation of hypothetic mechanism for BECN1 enriched MSC-ex mediating LX-2 ferroptosis through upregulating xCT/GPX4 signaling. MSC-ex can deliver BECN1 protein into HSCs and inhibits SLC7A11/xCT transcription in the nucleus. Decreased SLC7A11/xCT results in cysteine deficiency and GSH reduction in HSCs, leading to GPX4 reduction and ferroptosis. MSC-ex Mesenchymal stem cell derived exosome, xCT cystine/glutamate exchange transporter. GSH glutathione, r-glutamyl cysteingl glycine, GPX4 Glutathione peroxidase 4.

In summary, we demonstrated that MSC-ex targeted hepatic stellate cells activation in vitro and in vivo. This may be mediated by the delivery of BECN1 to induce ferroptosis via the downregulation xCT/GPX4 pathway (Fig. 7). These findings will provide different mechanisms for understanding the protective effect of MSC-ex against CCl_4_-induced liver fibrosis, and HSCs ferroptosis may be a new therapeutic approach for liver fibrosis treatment in the future.

## Supplementary information

Supplemental Figure 1

Supplemental Figure 2

Supplemental Figure 3

Supplemental Figure 4

Supplemental Figure 5

Supplemental Figure 6

Supplemental Fig. 7.

Supplemental Fig. 8.

Supplemental Fig. 9.

aj-checklist

Supplemental figure legends

## Data Availability

All data generated or used during the study appear in the submitted article.
