# Supplementary figures and images for: HucMSC-derived exosomes delivered BECN1 induces ferroptosis of hepatic stellate cells via regulating the xCT/GPX4 axis
Source: Cell Death Dis. 2022 Apr 8;13(4):319. doi: 10.1038/s41419-022-04764-2 (PMC8993870; doi:10.1038/s41419-022-04764-2)

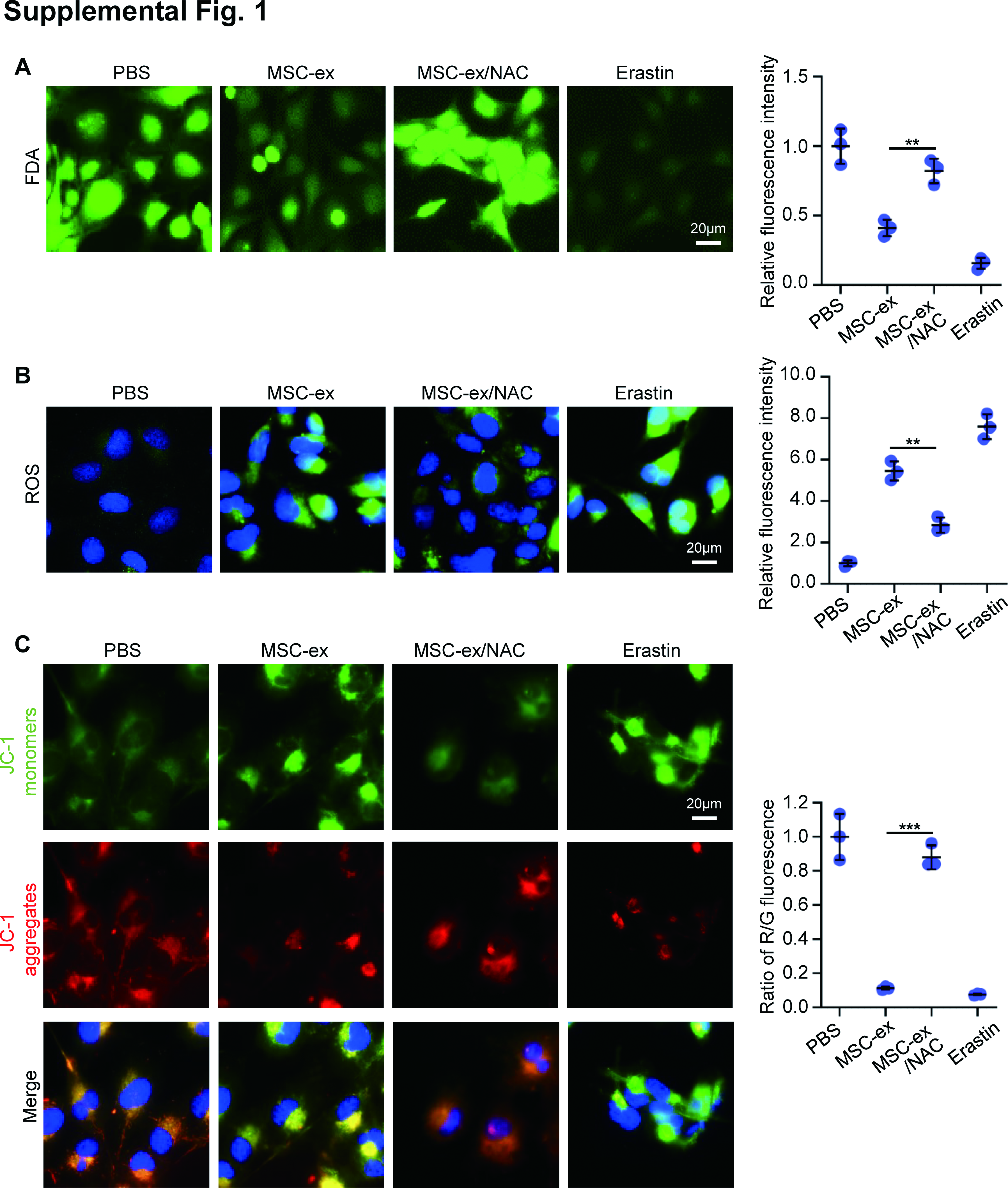

Supplement: Supplementary file 1 — Supplemental Figure 1 [file 41419_2022_4764_MOESM1_ESM.tif]

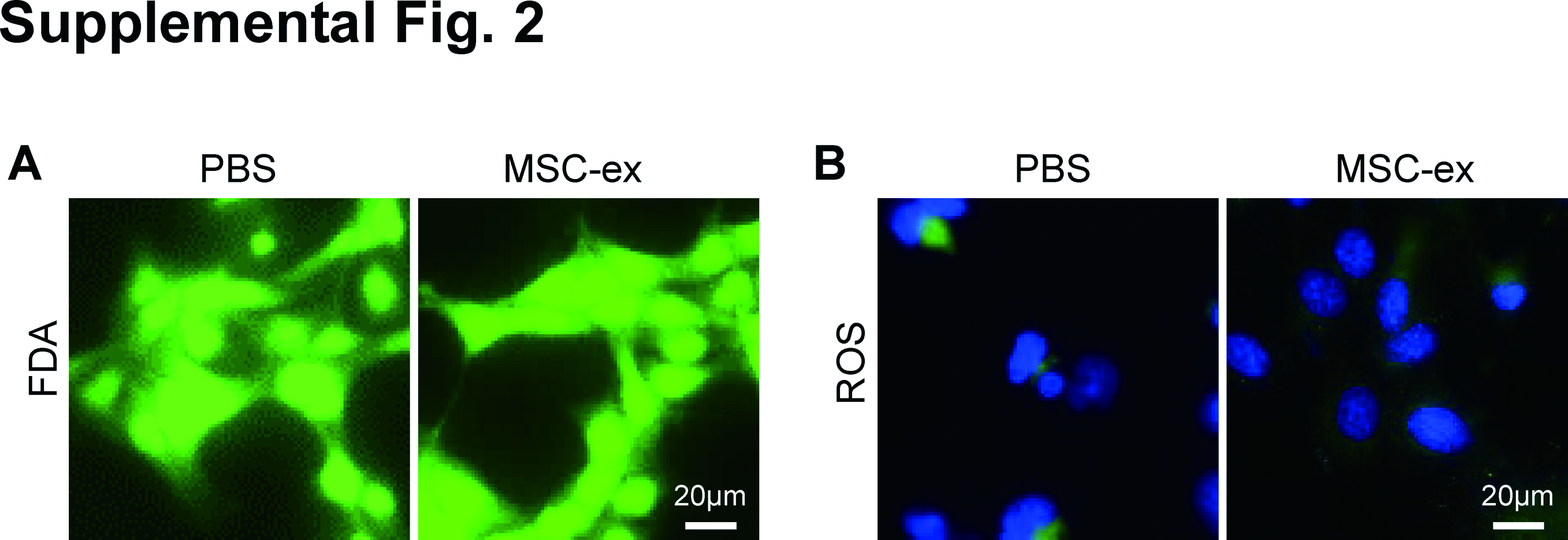

Supplement: Supplementary file 2 — Supplemental Figure 2 [file 41419_2022_4764_MOESM2_ESM.tif]

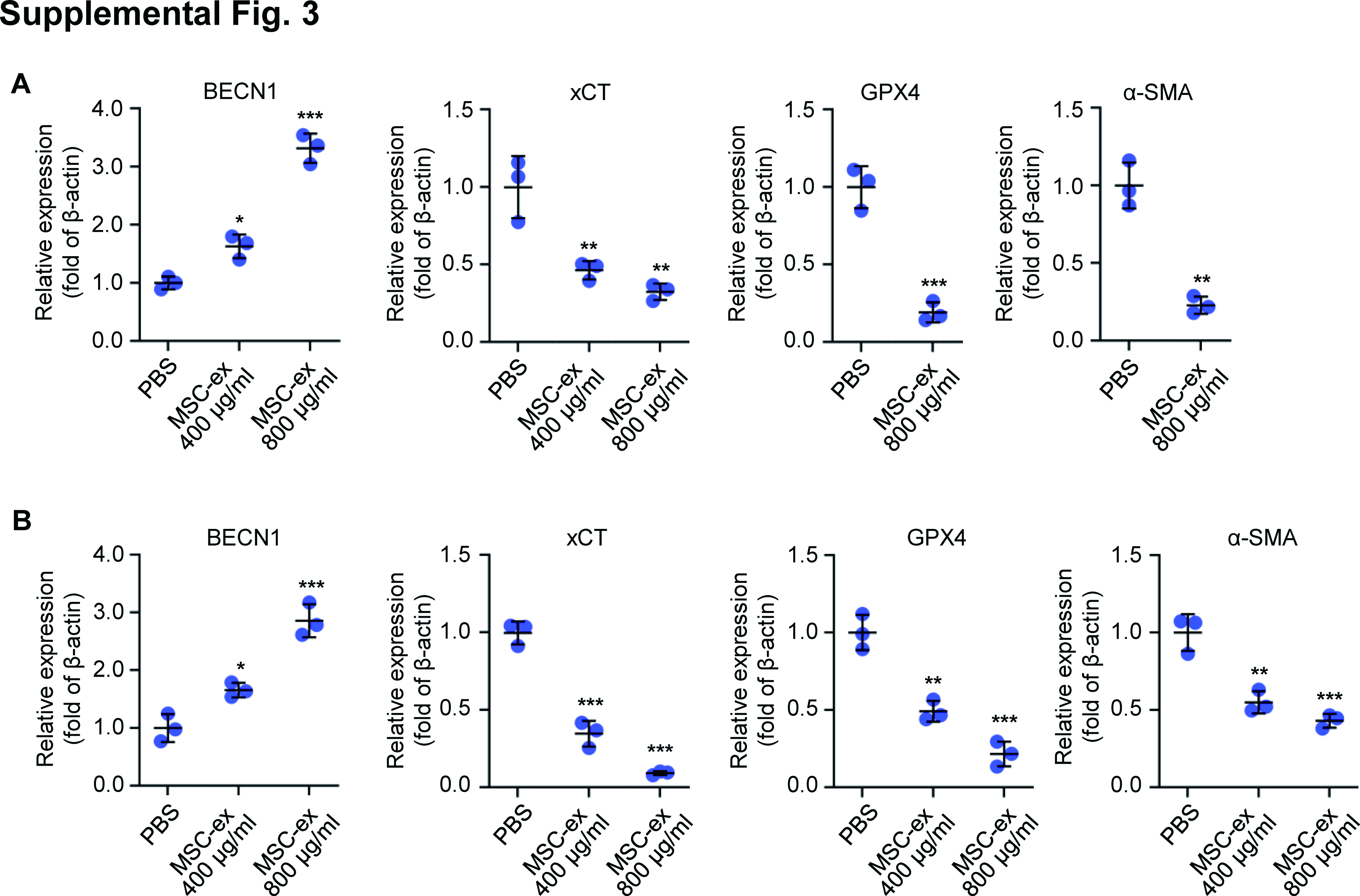

Supplement: Supplementary file 3 — Supplemental Figure 3 [file 41419_2022_4764_MOESM3_ESM.tif]

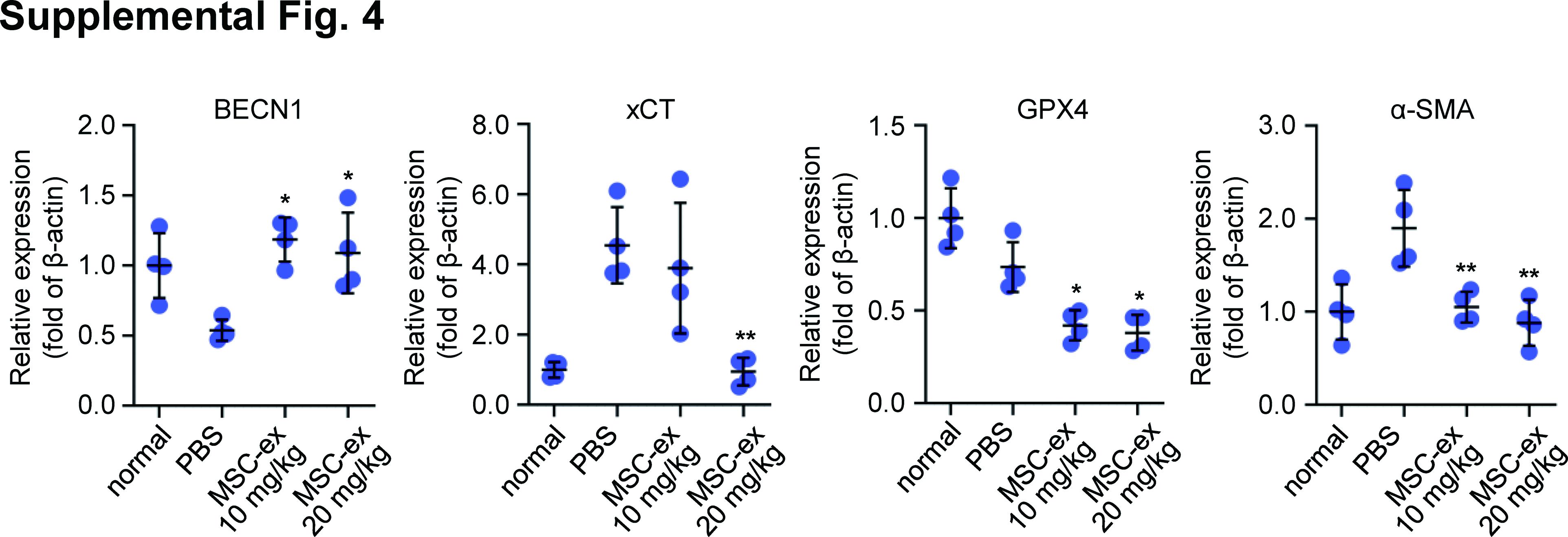

Supplement: Supplementary file 4 — Supplemental Figure 4 [file 41419_2022_4764_MOESM4_ESM.tif]

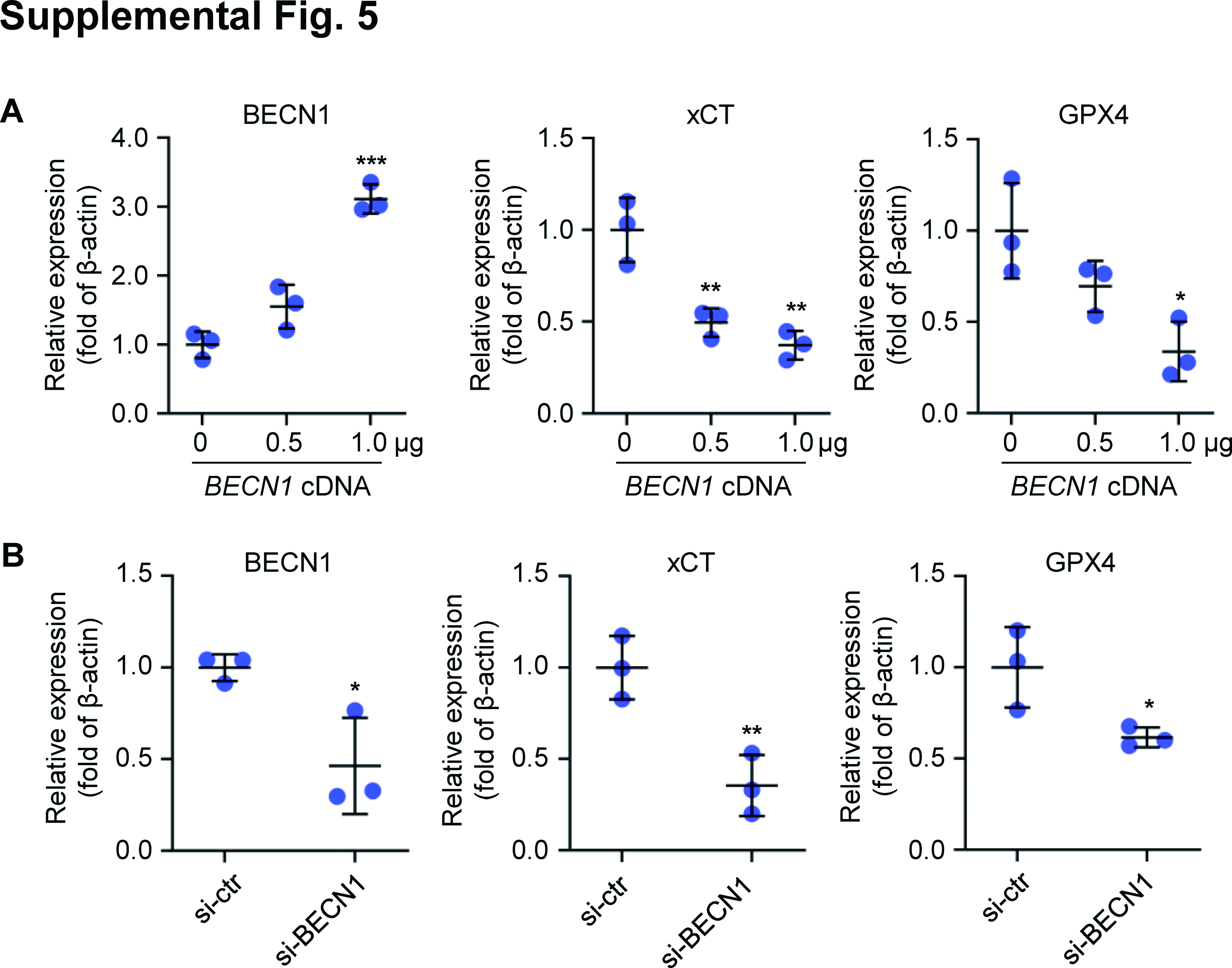

Supplement: Supplementary file 5 — Supplemental Figure 5 [file 41419_2022_4764_MOESM5_ESM.tif]

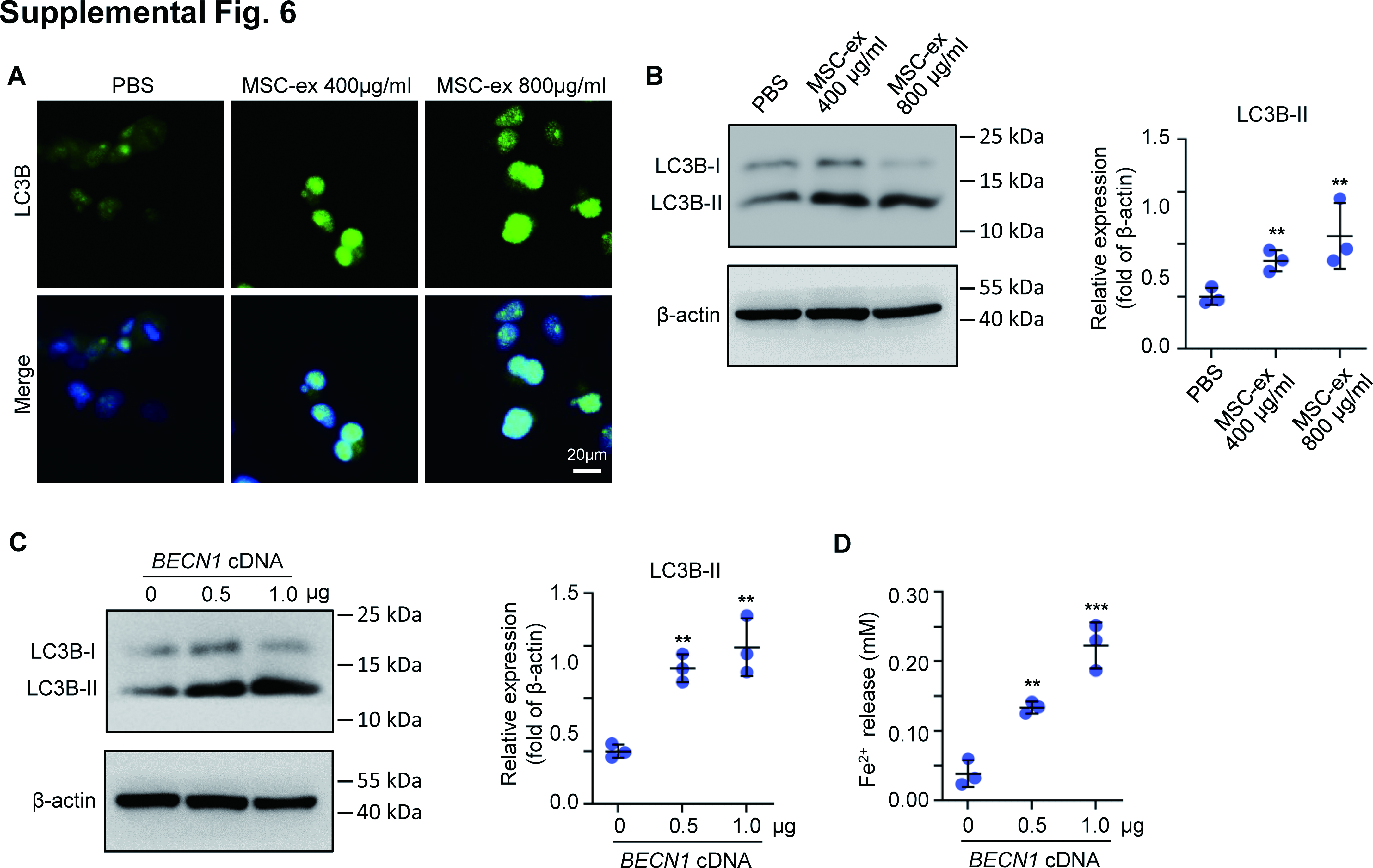

Supplement: Supplementary file 6 — Supplemental Figure 6 [file 41419_2022_4764_MOESM6_ESM.tif]

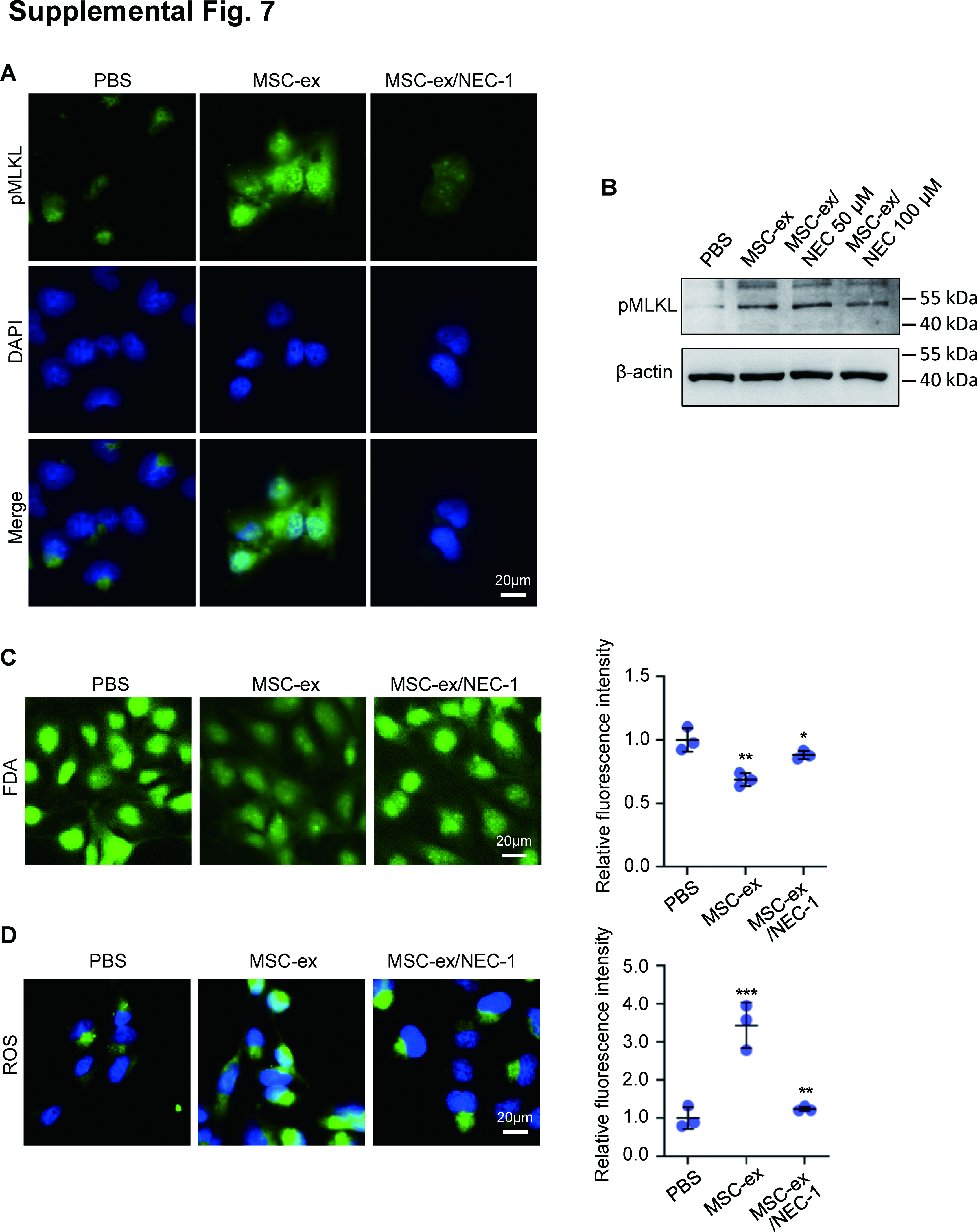

Supplement: Supplementary file 7 — Supplemental Fig. 7. [file 41419_2022_4764_MOESM7_ESM.tif]

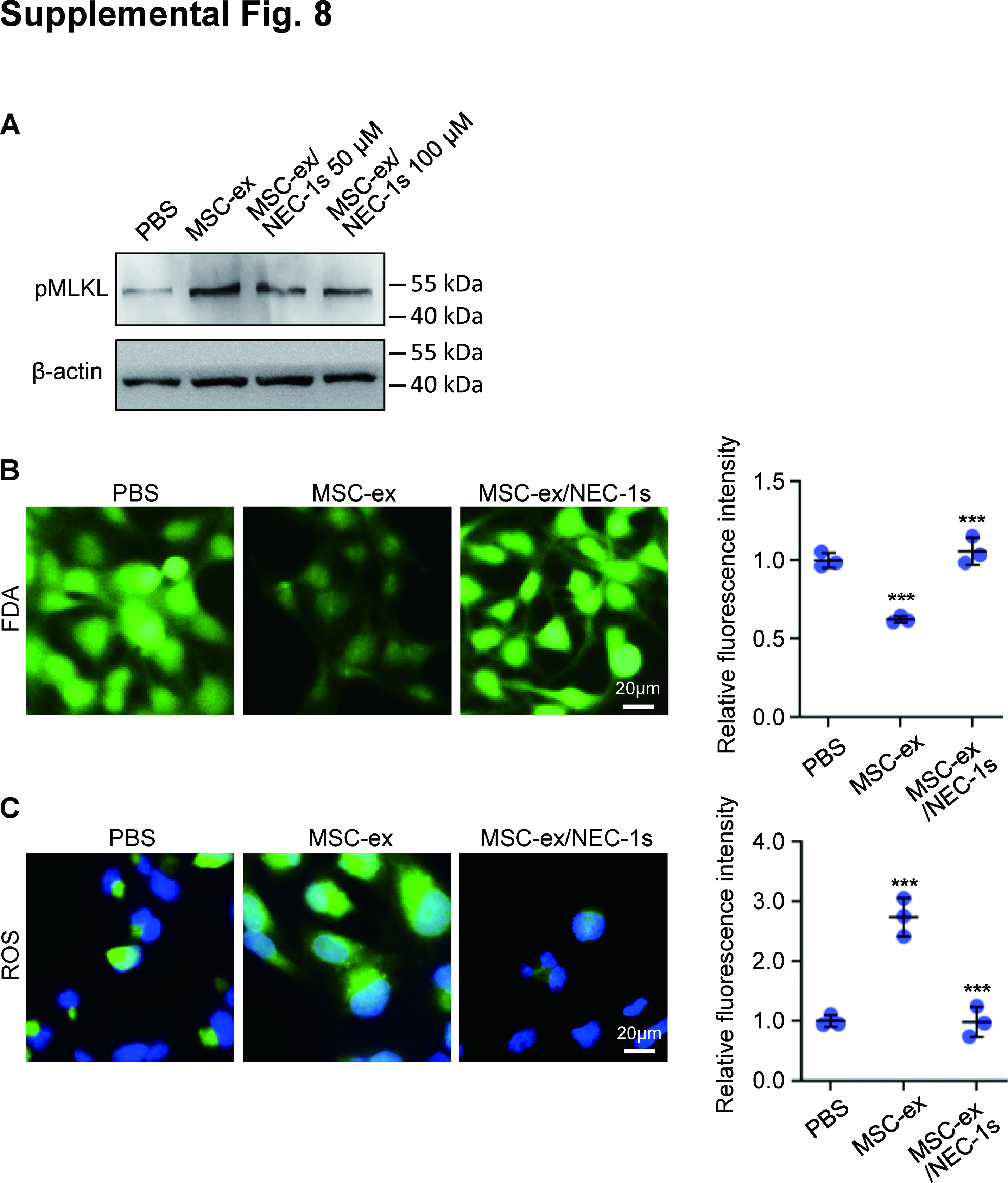

Supplement: Supplementary file 8 — Supplemental Fig. 8. [file 41419_2022_4764_MOESM8_ESM.tif]

A

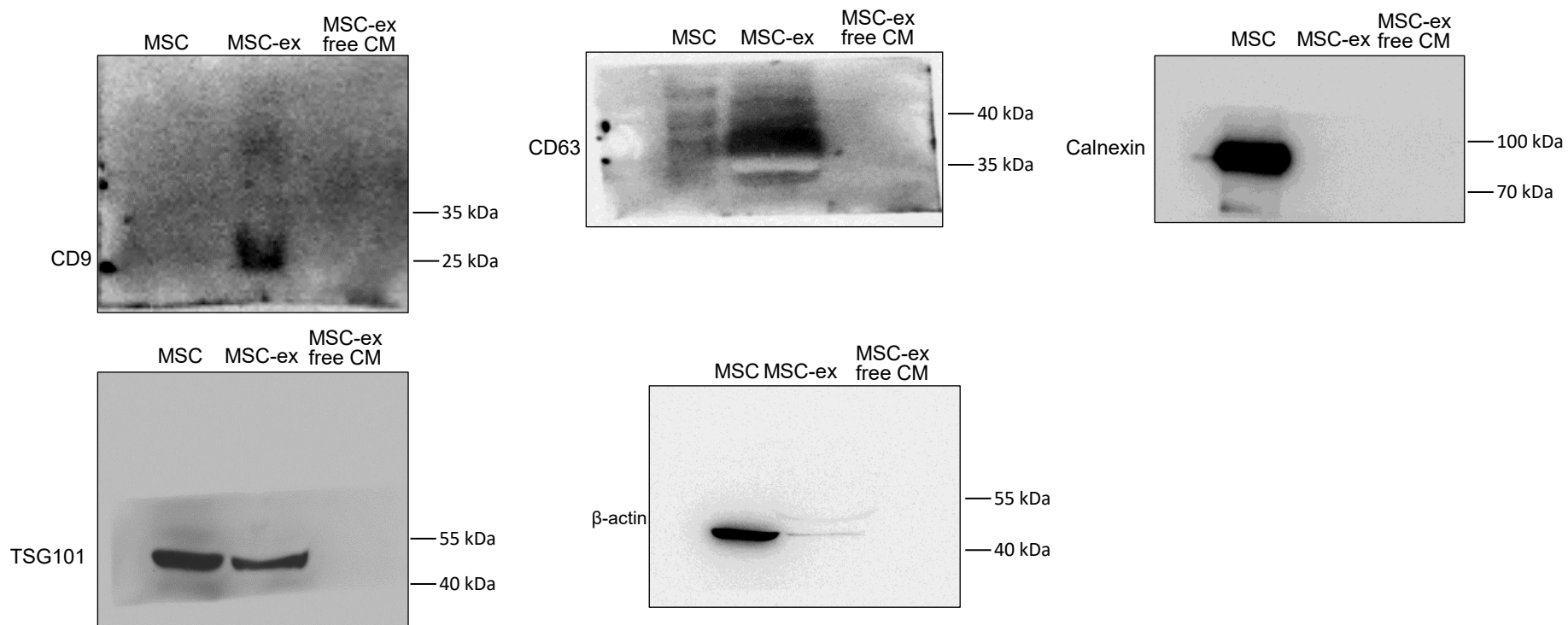

B

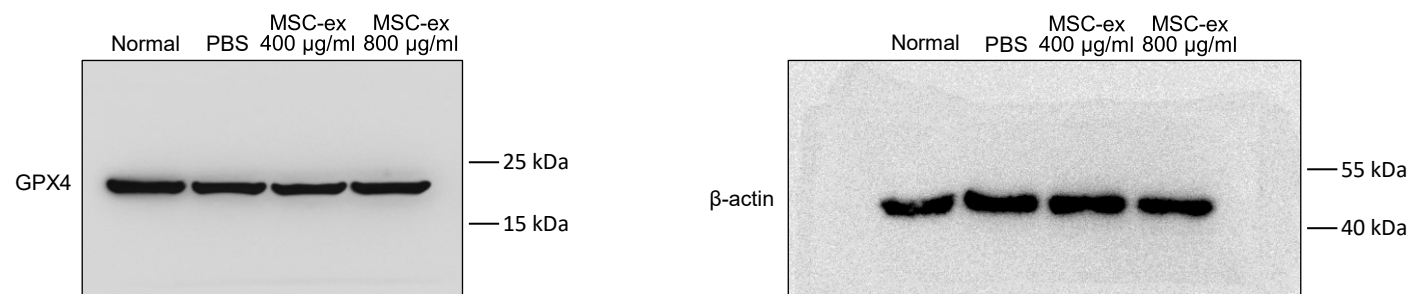

C

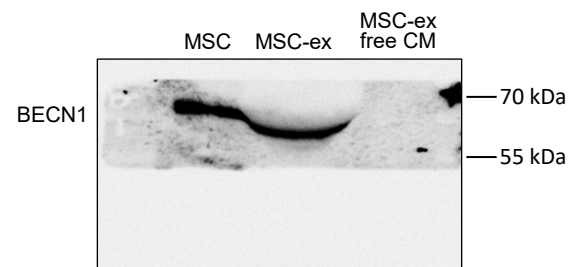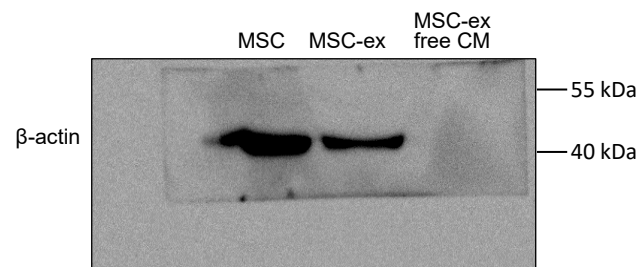

D

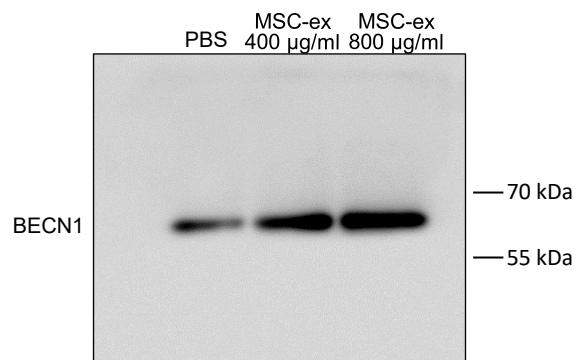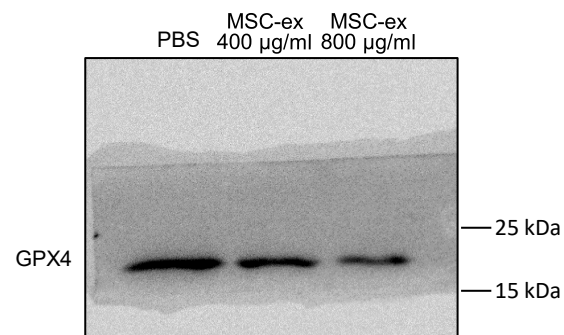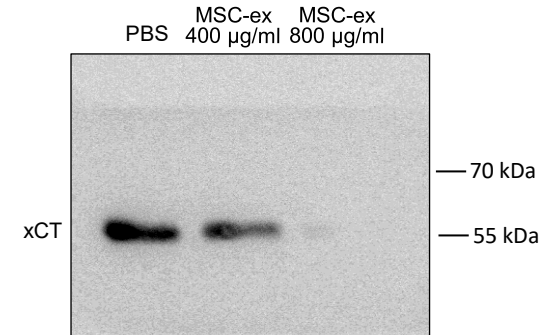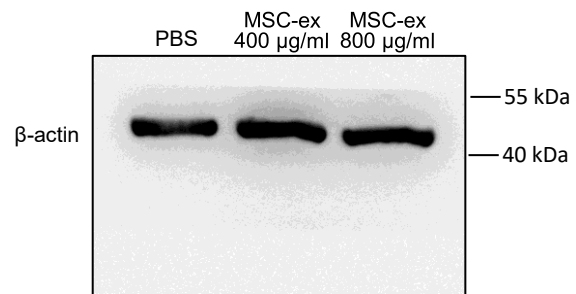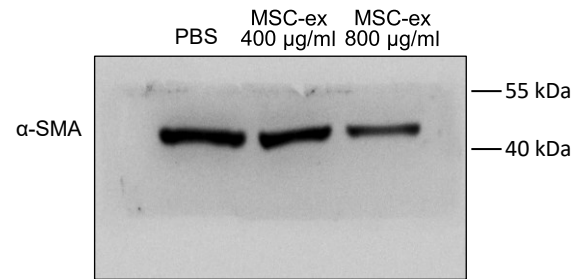

E

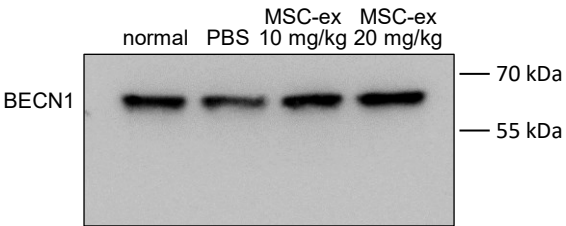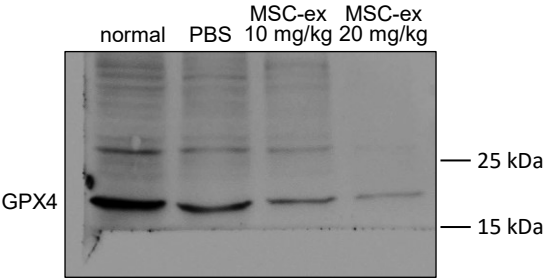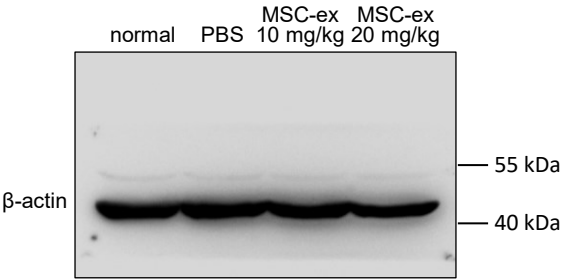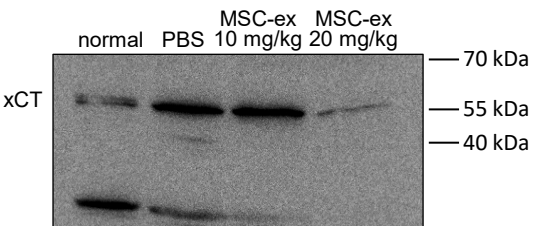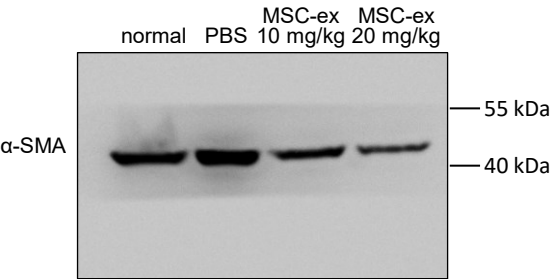

F

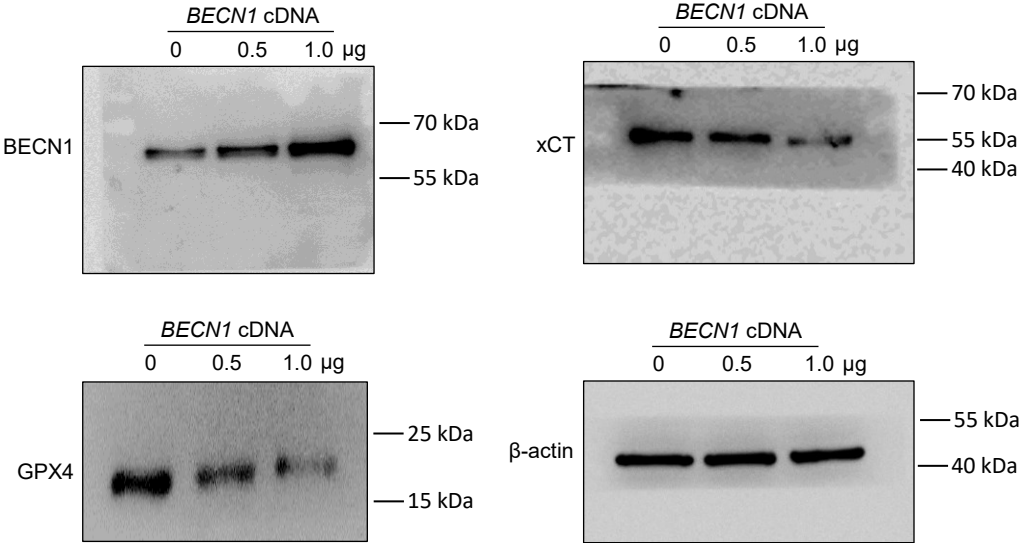

G

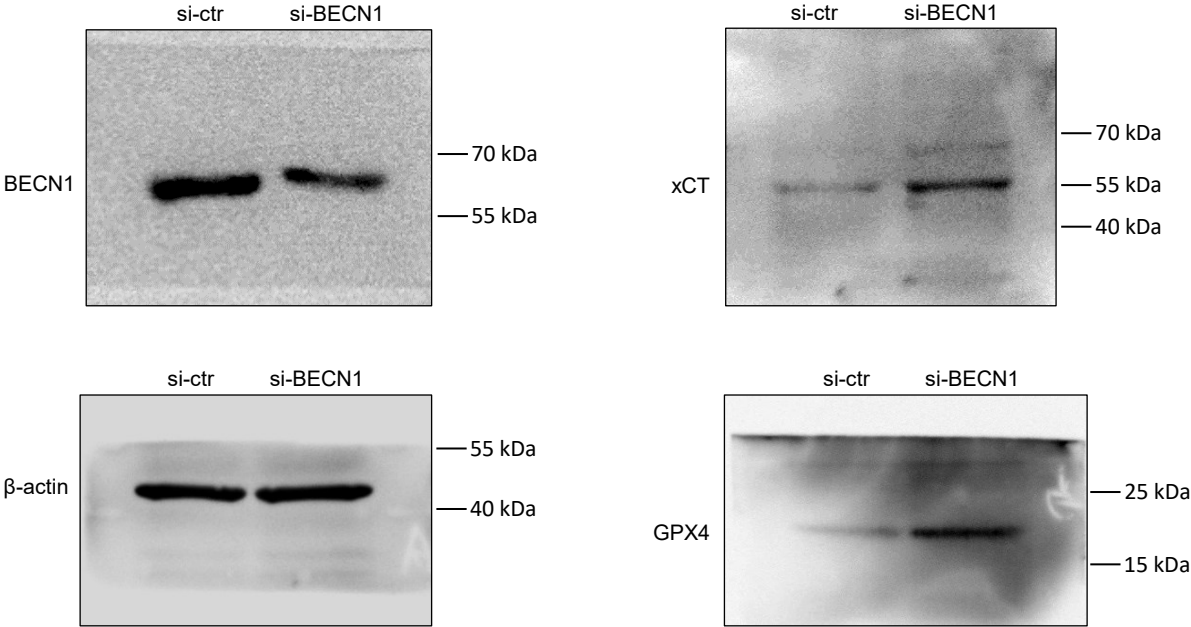

H

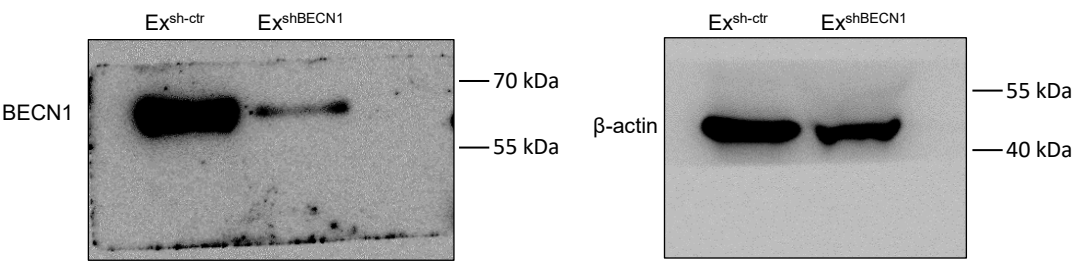

I

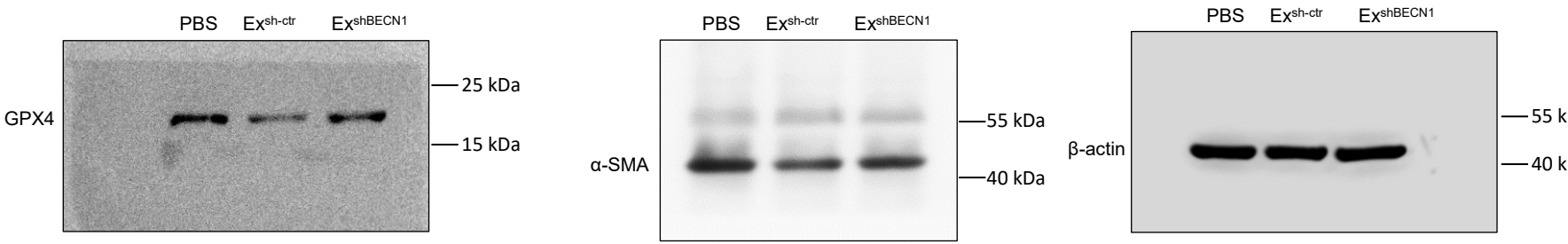

J

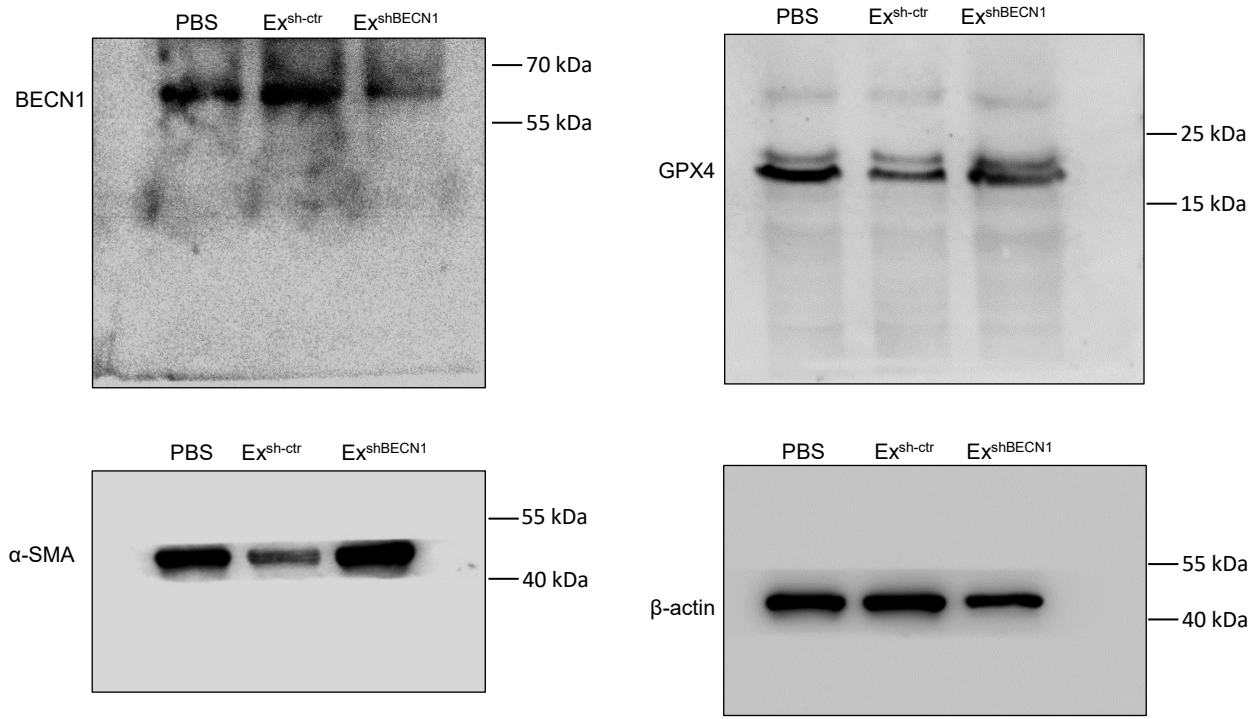

K

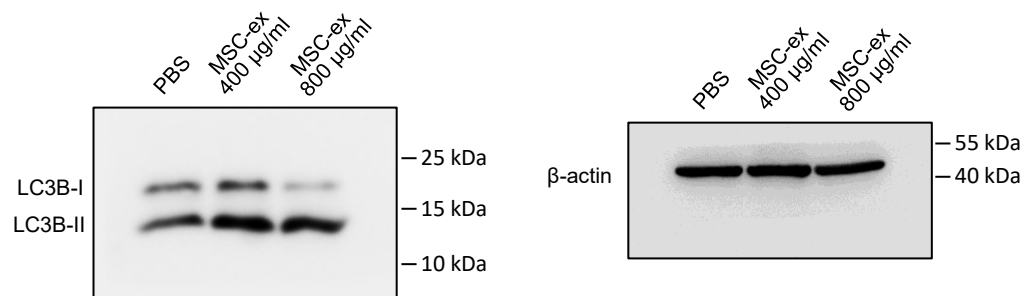

L

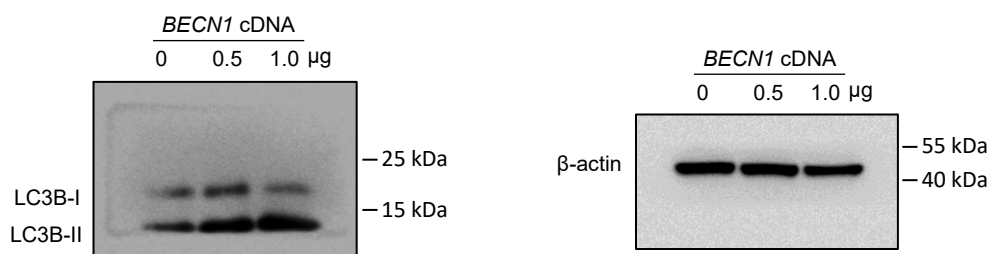

M

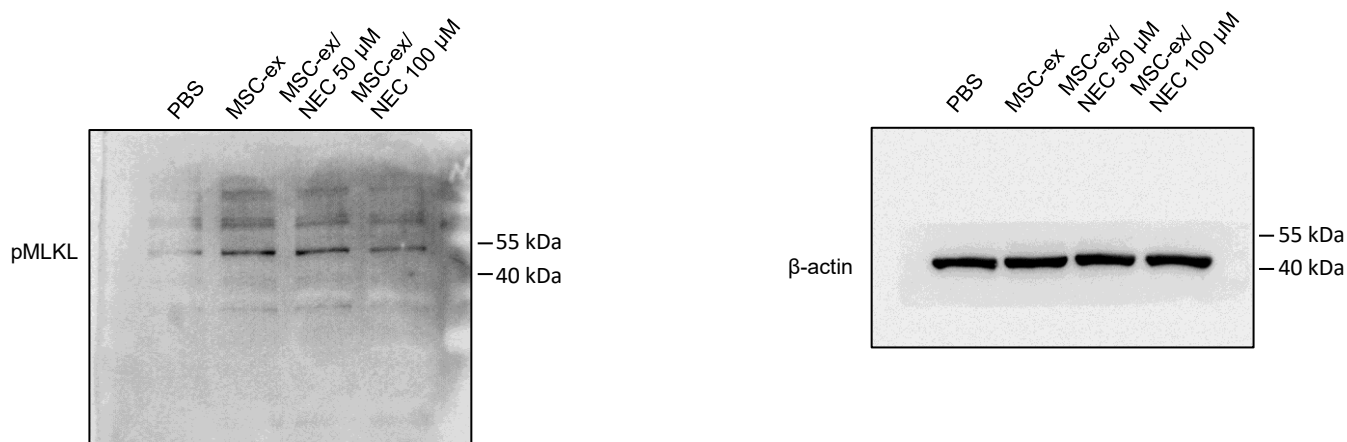

N

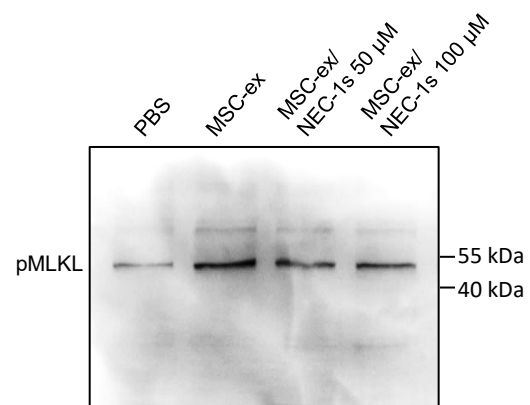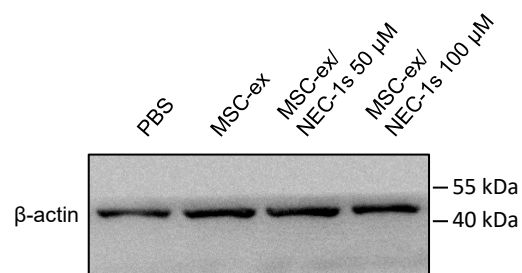

Supplement: Supplementary file 9 — Supplemental Fig. 9. [file 41419_2022_4764_MOESM9_ESM.pdf]
