# Supplementary material for: HucMSC-derived exosomes delivered BECN1 induces ferroptosis of hepatic stellate cells via regulating the xCT/GPX4 axis
Source: Cell Death Dis. 2022 Apr 8;13(4):319. doi: 10.1038/s41419-022-04764-2 (PMC8993870; doi:10.1038/s41419-022-04764-2)
Supplement: Supplementary file 11 — Supplemental figure legends [file 41419_2022_4764_MOESM11_ESM.docx]

Supplemental Fig. 1. NAC (N-acetyl-L-cysteine) reverted MSC-ex induced ferroptosis in LX-2.

(A) FDA staining of cell death in MSC-ex or MSC-ex/NAC treated LX-2 (n = 3; ** *P* < 0.01 compared with MSC-ex group). Scale bar, 20 μm. (B) ROS production in MSC-ex or MSC-ex/NAC treated LX-2 by DCF probe staining (n = 3; ** *P* < 0.01 compared with MSC-ex group). Scale bar, 20 μm. (C) Mitochondria membrane potential in MSC-ex or MSC-ex/NAC treated LX-2 by JC-1 staining (n = 3; *** *P* < 0.001 compared with MSC-ex group). Scale bar, 20 μm. MSC-ex, mesenchymal stem cell derived exosome. PBS, phosphate buffered saline. ROS, reactive oxygen species. MDA, malondialdehyde. GSH, glutathione, r-glutamyl cysteingl glycine. DCF, 2',7'-Dichlorofluorescein.

Supplemental Fig. 2. MSC-ex didn’t affect cell death and ROS production in HFL-1.

(A) FDA staining of cell death in MSC-ex treated HFL-1. Scale bar, 20 μm. (B) ROS production in MSC-ex treated HFL-1 by DCF probe staining. Scale bar, 20 μm. MSC-ex, mesenchymal stem cell derived exosome. PBS, phosphate buffered saline. FDA, Fluorescein Diacetate. ROS, reactive oxygen species. HFL-1, human embryonic lung fibroblast.

Supplemental Fig. 3. Quantitative analysis of MSC-ex delivered BECN1 upregulated System xc-/GPX4 axis in LX-2.

(A) Immunofluorescence quantification of BECN1, xCT, GPX4 and α-SMA protein expression in MSC-ex (400, 800 µg/ml) treated LX-2 (n = 3; * *P* < 0.05 compared with PBS group, ** *P* < 0.01 compared with PBS group, *** *P* < 0.001 compared with PBS group). Scale bars, 20 μm. (B) Western blot quantification of BECN1, xCT, GPX4 and α-SMA expression in MSC-ex (400, 800 µg/ml) treated LX-2 (n = 3; * *P* < 0.05 compared with PBS group, ** *P* < 0.01 compared with PBS group, *** P < 0.001 compared with PBS group). MSC-ex, mesenchymal stem cell derived exosome. PBS, phosphate buffered saline. GPX4, glutathione peroxidase 4. α-SMA, alpha smooth muscle actin. System xc-, cystine/glutamate antiporter system. xCT, cystine/glutamate exchange transporter.

Supplemental Fig. 4.  Quantitative analysis of MSC-ex upregulated BECN1/System xc-/GPX4 axis in mouse fibrotic liver.

Western blot quantification of BECN1, xCT, GPX4 and α-SMA expression in MSC-ex (10, 20 mg/kg) treated mouse liver (n = 4; * *P* < 0.05 compared with PBS group, ** *P* < 0.01 compared with PBS group). MSC-ex, mesenchymal stem cell derived exosome. PBS, phosphate buffered saline. GPX4, glutathione peroxidase 4. α-SMA, alpha smooth muscle actin. System xc-, cystine/glutamate antiporter system. xCT, cystine/glutamate exchange transporter.

Supplemental Fig. 5.  Quantitative analysis of BECN1 regulated LX-2 ferroptosis.

(A) Western blot quantification of BECN1, xCT and GPX4 expression in LX-2 transfected with 0.5 or 1.0 µg *BECN1* cDNA (n = 3; * *P* < 0.05 compared with 0 µg *BECN1* cDNA group, ** *P* < 0.01 compared with PBS group, *** *P* < 0.001 compared with 0 µg *BECN1* cDNA group). (B) Western blot quantification of BECN1, xCT and GPX4 expression in LX-2 transfected with control siRNA (si-ctr) or BECN1 siRNA (siBECN1) (n = 3; * *P* < 0.05 compared with si-ctr group, ** *P* < 0.01 compared with si-ctr group). MSC-ex, mesenchymal stem cell derived exosome. PBS, phosphate buffered saline. GPX4, glutathione peroxidase 4. System xc-, cystine/glutamate antiporter system. xCT, cystine/glutamate exchange transporter.

Supplemental Fig. 6.  MSC-ex upregulated LC3B expression in LX-2.

(A) Immunofluorescence of LC3B protein expression in MSC-ex (400, 800 µg/ml) treated LX-2. (B) Western blot analysis of LC3B protein expression in MSC-ex (400, 800 µg/ml) treated LX-2 (n = 3; ** *P* < 0.01 compared with PBS group). (C) Western blot analysis of LC3B protein expression in LX-2 transfected with 0.5 or 1.0 µg *BECN1* cDNA (n = 3; ** *P* < 0.01 compared with 0 µg *BECN1* cDNA group). (D) Fe2+ level in LX-2 transfected with 0.5 or 1.0 µg *BECN1* cDNA (n = 3; ** *P* < 0.01 compared with PBS group, *** *P* < 0.001 compared with 0 µg *BECN1* cDNA group). MSC-ex, mesenchymal stem cell derived exosome. PBS, phosphate buffered saline.

Supplemental Fig. 7. NEC-1 (necrostatin-1) diminish MSC-ex induced ferroptosis in LX-2.

(A) Immunofluorescence of pMLKL protein expression in MSC-ex or MSC-ex/NEC-1 treated LX-2. (B) Western blot analysis of pMLKL protein expression in MSC-ex or MSC-ex/NEC-1 treated LX-2. (C) FDA staining of cell death in MSC-ex or MSC-ex/NEC-1 treated LX-2 (n = 3; * *P* < 0.05 compared with PBS group, ** *P* < 0.01 compared with PBS group). Scale bar, 20 μm. (D) ROS production in MSC-ex or MSC-ex/NEC-1 treated LX-2 by DCF probe staining (n = 3; ** *P* < 0.01 compared with PBS group, *** *P* < 0.001 compared with PBS group). Scale bar, 20 μm. MSC-ex, mesenchymal stem cell derived exosome. PBS, phosphate buffered saline. FDA, Fluorescein Diacetate. ROS, reactive oxygen species.

Supplemental Fig. 8. NEC-1s diminish MSC-ex induced ferroptosis in LX-2.

(A) Western blot analysis of pMLKL protein expression in MSC-ex or MSC-ex/NEC-1s treated LX-2. (B) FDA staining of cell death in MSC-ex or MSC-ex/NEC-1s treated LX-2 (n = 3; *** *P* < 0.001 compared with PBS group). Scale bar, 20 μm. (C) ROS production in MSC-ex or MSC-ex/NEC-1 treated LX-2 by DCF probe staining (n = 3; *** *P* < 0.001 compared with PBS group). Scale bar, 20 μm. MSC-ex, mesenchymal stem cell derived exosome. PBS, phosphate buffered saline. FDA, Fluorescein Diacetate. ROS, reactive oxygen species.
